# A novel transgenic zebrafish line for red opsin expression in outer segments of photoreceptor cells

**DOI:** 10.1002/dvdy.24631

**Published:** 2018-04-23

**Authors:** Cátia Crespo, Daniele Soroldoni, Elisabeth Knust

**Affiliations:** ^1^ Max Planck Institute of Molecular Cell Biology and Genetics Dresden Germany; ^2^ École Polytechnique Fédérale de Lausanne Lausanne Switzerland

**Keywords:** Development, retina, cone cells, LWS1, LWS2

## Abstract

**Background:** Opsins are a group of light‐sensitive proteins present in photoreceptor cells, which convert the energy of photons into electrochemical signals, thus allowing vision. Given their relevance, we aimed to visualize the two red opsins at subcellular scale in photoreceptor cells. **Results:** We generated a novel Zebrafish BAC transgenic line, which express fluorescently tagged, full‐length Opsin 1 long‐wave‐sensitive 1 (Opn1lw1) and full‐length Opsin 1 long‐wave‐sensitive 2 (Opn1lw2) under the control of their endogenous promoters. Both fusion proteins are localized in the outer segments of photoreceptor cells. During development, Opn1lw2‐mKate2 is detected from the initial formation of outer segments onward. In contrast, Opn1lw1‐mNeonGreen is first detected in juvenile Zebrafish at about 2 weeks postfertilization, and both opsins continue to be expressed throughout adulthood. It is important to note that the presence of the transgene did not significantly alter the size of outer segments. **Conclusions:** We have generated a transgenic line that mimics the endogenous expression pattern of Opn1lw1 and Opn1lw2 in the developing and adult retina. In contrast to existing lines, our transgene design allows to follow protein localization. Hence, we expect that these lines could act as useful real‐time reporters to directly measure phenomena in retinal development and disease models. *Developmental Dynamics 247:951–959, 2018*. © 2018 The Authors Developmental Dynamics published by Wiley Periodicals, Inc. on behalf of American Association of Anatomists

## Key Findings

- We have generated multiple transgenes that mimic the endogenous expression pattern of the two red opsins, Opn1lw1 and Opn1lw2, in the developing and adult retina.
- The opsin fusion protein reflects the endogenous proteins dynamics.
- This line provides a tool for various applications, including the analysis of expression, protein localisation and trafficking and opsin switching.

## Introduction

Photoreceptor cells (PRCs) are specialized neurons that, in vertebrates, are classified into rods, specialized for dim‐light vision, and cones (Mustafi et al., 2009), responsible for bright‐light and color vision (Carter‐Dawson and Lavail, 1979; Kawamura and Tachibanaki, 2008). All PRCs are characterized by a pronounced apicobasal polarity, with the apical domain subdivided into the inner segment (IS) and the outer segment (OS). The OS is a modified primary cilium essential for phototransduction. The OS of developing and mature PRCs is characterized by a tremendously expanded apical membrane in order to accommodate the huge amount of visual pigment (reviewed in May‐Simera et al., 2017). The visual pigment consists of a transmembrane protein, opsin, linked to a chromophore, which is responsible for absorption of photons of light (Allison et al., 2004; Enright et al., 2015; Samardzija et al., 2009; Saszik and Bilotta, 1999; Suliman and Novales Flamarique, 2014; Yokoyama, 2000). Correct opsin localization in the OS requires active transport of the protein from the cell body into the OS and is essential for PRC function and survival (reviewed in Bales and Gross, 2016; Hollingsworth and Gross, 2012).

In Zebrafish, opsins have been classified into five different groups according to the wavelength they absorb. Due to genome duplications, 10 different opsin genes are encoded in the Zebrafish genome: two rhodopsin genes (Morrow et al., 2017), one blue and one ultraviolet opsin gene, four different green opsin genes, and two red opsin genes. Opsin genes are expressed in different PRC subtypes. Rhodopsin is found in rod PRCs. Cones can be classified into four different subtypes according to the opsin they express, namely the ultraviolet‐, blue‐, red‐, and green‐sensitive cones (Branchek and Bremiller, 1984; Chinen et al., 2003; Minamoto and Shimizu, 2005).

In Zebrafish, the two red opsin genes (*opn1lw1* and *opn1lw2*) are found in a tandem array (head to tail) on the same chromosome, and it was shown that they share their regulatory elements (Tsujimura et al., 2010). This locus was used to study the transcriptional regulation of tandemly replicated opsins to demonstrate that expression can switch from one to the other paralog, which was referred to as opsin switching (Mitchell et al., 2015). Furthermore, red opsins are expressed in one of the most abundant cone PRCs.

Several Zebrafish reporter lines have been generated in the past, which allow discrimination of the different PRC subtypes. In most cases, endogenous promoters drive the expression of fluorescent proteins, which replace or disrupt the coding sequence of the respective gene of interest (DuVal et al., 2014; Fadool, 2003; Fang et al., 2017; Fang et al., 2013; Fraser et al., 2013; Hagerman et al., 2016; Hamaoka et al., 2002; Kennedy et al., 2007; Kennedy et al., 2001; Li et al., 2012; Luo et al., 2004; Ogawa et al., 2015; Raymond et al., 2014; Suzuki et al., 2013; Takechi et al., 2008; Takechi et al., 2003, Tsujimura et al., 2010; Tsujimura et al., 2007; Yu et al., 2007; Zou et al., 2010). Therefore, these transgenic (TG) lines can be considered transcriptional reporters, which can be used to trace the onset of transcription and track cells that express the transgene, but fail to report the dynamics of (protein) expression due to the prolonged stability of the fluorescent protein. In addition, proteins expressed from these reporter lines typically lack functional opsin domains, which are required for proper protein (subcellular) localization and stability (Mitchell et al., 2015). Thus, fluorescent proteins are restricted to the cytoplasm of PRCs and are not actively transported to the OS.

To visualize the OS of rod PRCs, a transgenic Zebrafish line has been established, which encodes a *Xenopus* Rhodopsin‐green fluorescent protein (GFP) fusion protein, expressed under the control of the *Xenopus* rhodopsin promoter (Perkins et al., 2002; Tam et al., 2000). This line overcomes the technical limitations mentioned above, but it can be used only to study rods (Perkins et al., 2002), the best studied PRCs to date. Cones, on the other hand, which are essential for color vison, have received less attention so far. The Zebrafish retina contains predominantly cones, and the most abundant cones are those that express the red opsins. This makes *opn1lw1* and *opn1lw2* ideal candidates to study cone OS maturation and maintenance (Raymond et al., 1995).

In this study, we generated a new bacterial artificial chromosome (BAC)‐based transgene that comprises the endogenous loci of the two red opsins, *opn1lw1* and *opn1lw2*. The transgene was designed to express opsin fusion proteins, which can be transported to the OS. This line now distinguishes the expression and localization of both red opsins during maturation and maintenance of the OS. Furthermore, this transgenic line has the potential to be used as real‐time reporter to analyze OS formation and degeneration, opsin trafficking, and opsin switching.

## Results and Discussion

### Generation of a Transgenic Line Expressing the Two Red Opsins

**Figure 1:**
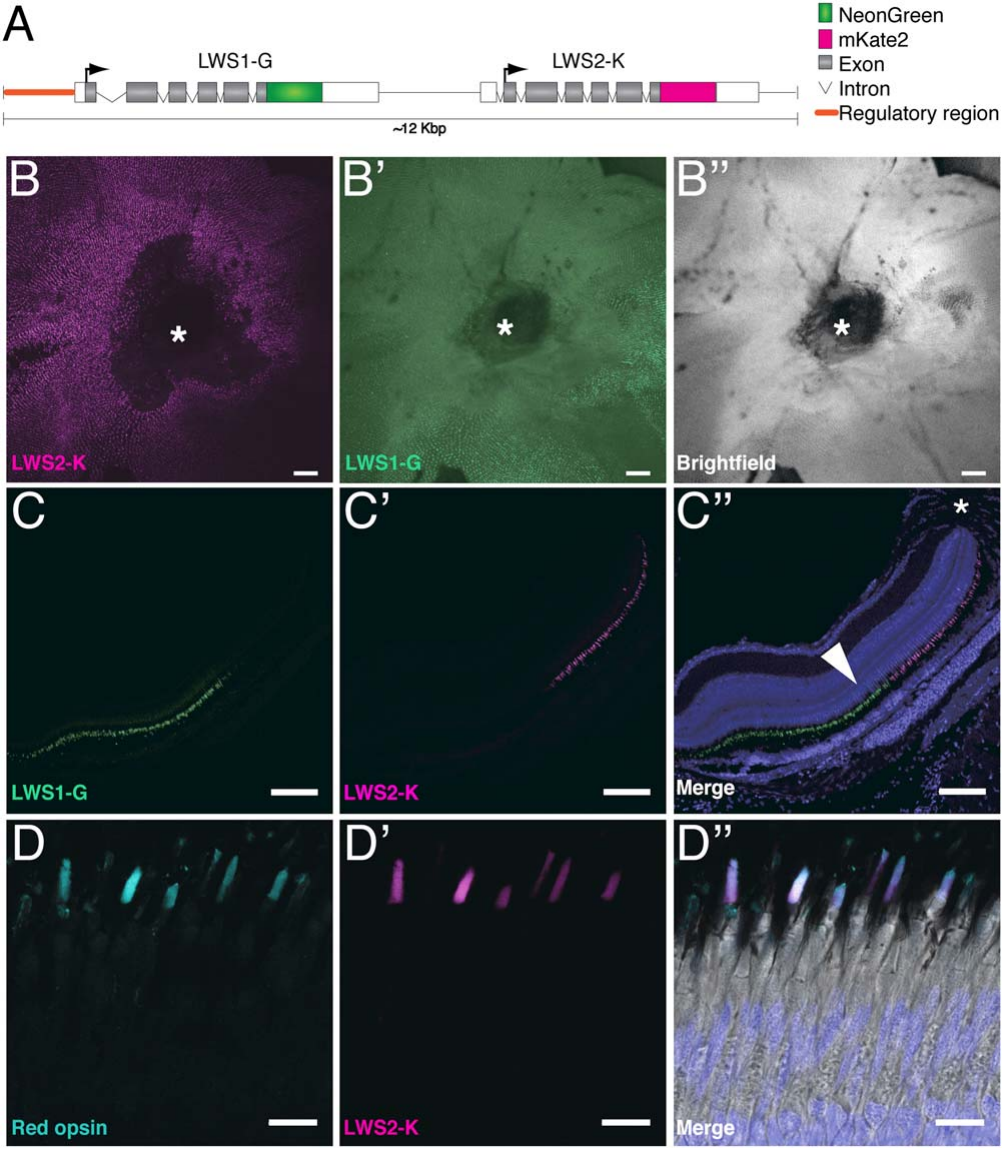
LWS1‐G and LWS2‐K are detected in outer segment of photoreceptor cells. **A**: Schematic illustration of Tg(LWS) (∼12 Kb, not to scale). Opn1lw1 and Opn1lw2 are C‐terminally fused to mNeonGreen and mKate2, respectively. The regulatory region (orange line) as defined by Tsujimura et al. (2010). The arrow depicts the orientation of the ORF. **B–B″**: Flat‐mounted retina of Tg(LWS) with LWS2‐K in magenta, LWS1‐G in green, and brightfield in gray. Asterisks outline the optic nerve of the retina. **C–C″**: Retinal section of an adult Tg(LWS) transgenic fish, ventral to the optic nerve (asterisk in C″). C″ shows the merge of C, C′, and DAPI staining (blue). Fluorescence of LWS1‐G is marked in green and LWS2‐K in magenta. Arrowhead in C″ highlights the outer nuclear layer. **D–D″**: Section of a Tg(LWS) adult retina showing staining with a red opsin antibody (cyan, D, D″) and fluorescence of LWS2‐K (magenta, D′, D″). D″ shows the merge of red opsin antibody staining (cyan), LWS2‐K fluorescence (magenta), DAPI staining (blue), and brightfield images. Scale bars B–C″ = 100 μm. Scale bars D–D″ = 10 μm.

To study the OS of cones, we decided to focus our attention on red‐sensitive cones, which are among the most abundant in the Zebrafish retina. To this end, we employed BAC recombineering to embed fluorescent reporter cassettes into the red opsin loci, *opn1lw1* and *opn1lw2,* which are arranged head to tail on the same chromosome (Fig. 1A). The BAC contained all regulatory regions, which are shared by the two loci (Allison et al. 2010; Salbreux et al. 2012; Chinen et al. 2003; Branchek and Bremiller 1984; Minamoto and Shimizu 2005). Unlike previously published transgenes, in which the coding regions are disrupted (Mitchell et al., 2015; Tsujimura et al., 2010), we replaced the stop codons of *opn1lw1* and *opn1lw2* with mNeonGreen and mKate2, respectively, which resulted in two intact C‐terminal fusion proteins. This approach aimed to exploit all regulatory cues, harbored in the endogenous loci, which are required to mimic reporter transcription, translation, and stability. The latter is essential for the “temporal resolution” of transgene expression and needs to be considered if a transgene should be used as a real‐time reporter.

To avoid overexpression of untagged open reading frames present on the BAC and to increase the transgenesis frequency, which is typically very low for large transgene constructs, we introduced a second recombination step to subclone the modified region of interest into a plasmid backbone containing I‐SceI Meganuclease sites. This “shaved” BAC (approx. 12 kb in size) included the 2.6‐kb upstream region of *opn1lw1* (Fig. 1A), which was shown to be required and sufficient to drive *opn1lw1* and *opn2lw2* transcription (Tsujimura et al., 2010).

The resulting construct was co‐injected with I‐SceI Meganuclease into Zebrafish embryos. All injected embryos were raised until adulthood and crossed with wild‐type (WT) animals. Their progeny was screened at 5 days postfertilization (dpf) with a fluorescent stereomicroscope, and five out of the seven screened clutches showed progeny that was mKate2‐positive. At this stage, Opn1lw2‐mKate2 (longwavelength‐sensitive 2‐mKate2, LWS2‐K) was visible in live embryos in the retina and the pineal gland, but Opn1lw1‐mNeonGreen (longwavelength‐sensitive 1‐mNeonGreen, LWS1‐G) could not be detected. We compared the progenies of five independent transgenic founders and did not find obvious differences in the onset of LWS2‐K fluorescence, intensity, or spatial distribution of the fusion protein. Thus, all further studies were performed with the progeny of one transgenic founder, designated as TgBac (‐2.6opn1lw1:opn1lw1‐mNeonGreen/opn1lw2‐mKate)^cbg9Tg^ (ZFIN; https://zfin.org/ZDB-ALT-180320-10), which we will refer to as Tg(LWS). To test the stability of the transgene insertion, transgenic animals were continuously outcrossed with WT fish until the F4 generation was obtained. LWS2‐K expression was detected throughout all generations, indicating that the transgene was stably inserted in the Zebrafish genome.

### Localization of Transgene‐expressed Protein in Outer Segments

As stated earlier, LWS2‐K could be detected in the early stages of the Zebrafish retina, indicating the presence and activity of the transgene. However, LWS1‐G could not be detected. To investigate if LWS1‐G is active at all and to see if LWS2‐K fluorescence is maintained, we examined retinas of adult transgenic fish. Flat‐mounted adult retina clearly showed the activity of both transgenes (Fig. 1B–B″). To further study the subcellular localization of both fusion proteins, we performed cryosections and imaged the fluorescence of fusion proteins without any further amplification of the signal in fixed tissues. In Tg(LWS) adult retinas, both LWS1‐G and LWS2‐K were restricted to the PRC layer, distal to the outer nuclear layer (Fig. 1C–C″). In accordance with previous reports (Takechi and Kawamura, 2005; Tsujimura et al., 2010), the number of LWS1‐G‐positive cells was highest in the ventral‐most region, with a few positive cells on the dorsal side, while LWS2‐K was restricted to the central region in the adult retina (Fig. 1B–C″). Some LWS1‐G‐positive cells could be detected in the central retina, and some LWS2‐K were found in the peripheral retina (Fig. 1B–C″).

To further confirm the subcellular localization of the fusion proteins in the OS, we conducted immunohistochemistry with a polyclonal antibody raised against Zebrafish red opsin. This antibody was shown to recognize red opsin proteins in the retina and a protein of the predicted size in western blots (Tsujimura et al., 2010; Vihtelic et al., 1999). Retinal sections of adult WT and Tg(LWS) siblings were prepared and processed in parallel (on the same slide) to control the immunohistochemistry protocol. In Tg(LWS), we found that the fluorescence of LWS2‐K and red opsin antibody signal largely overlaps in the OS of PRCs (Fig. 1D–D″). At first, this correlation was not surprising since the polyclonal antibody could recognize the endogenous and transgenic protein. However, we could not detect a significantly stronger signal when we compared retinal sections of WT and TG littermates stained with the antibody on the same slide. This raises the interesting possibility that the amount of opsin present in the OS is regulated. However, not all antibody‐positive cells showed reporter fluorescence. This could have various reasons: Fixation of the sample could affect protein structure, which can impair fluorescence intensity (Kusser and Randall 2003). Alternatively, transgene expression could be silenced in some LWS cones. The discrepancy between the antibody staining and the fluorescence could be resolved by further experiments using monoclonal antibodies raised against red opsins.

### LWS1‐G and LWS2‐K are Differentially Expressed During Retinal Development

**Figure 2:**
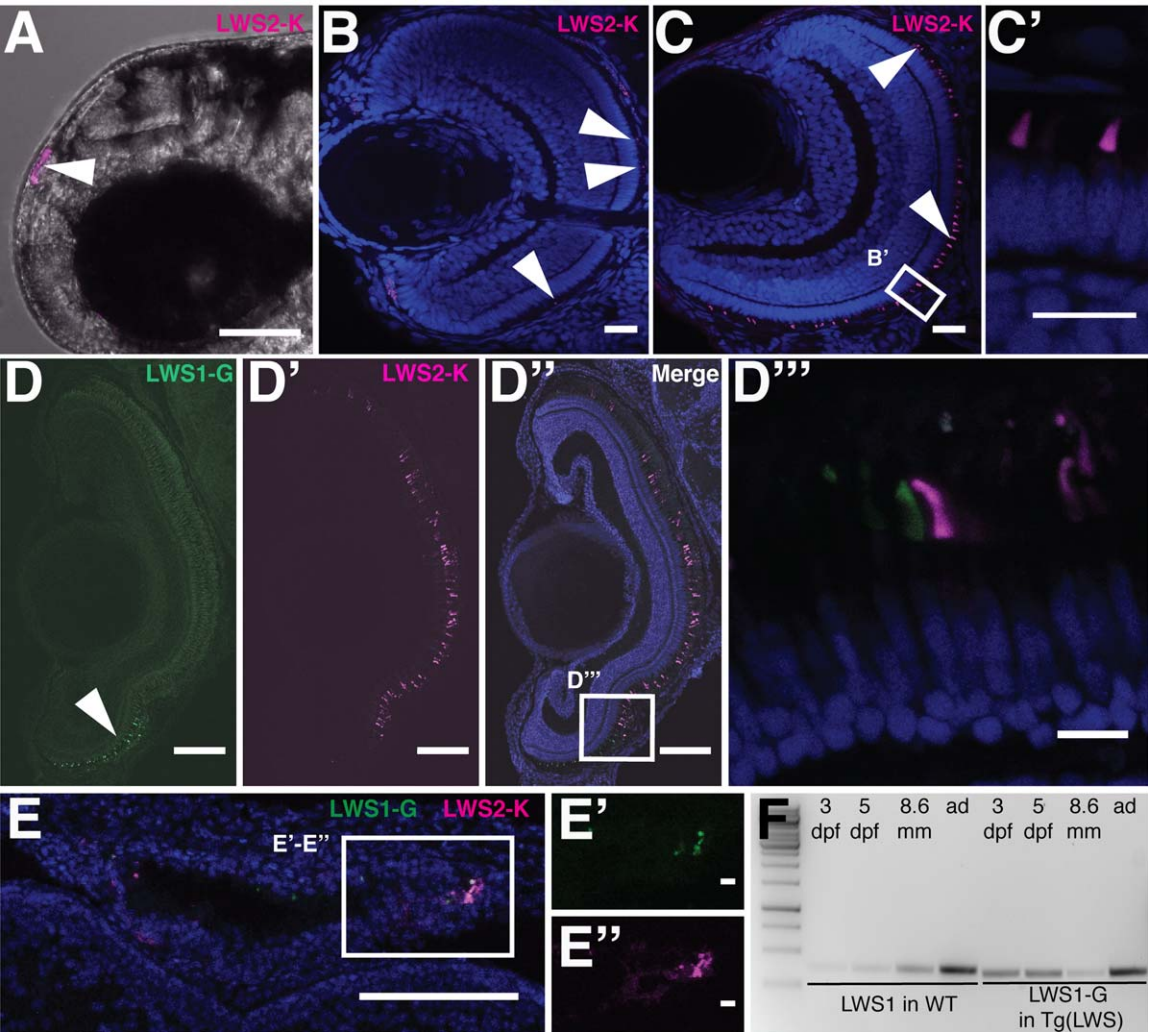
LWS1‐G and LWS2‐K protein expression during Zebrafish retinal development. **A:** LWS2‐K expression in the pineal gland (arrowhead) of a live Zebrafish embryo at 2 dpf. **B–C:** Retinal section of Tg(LWS) at 3 dpf with LWS2‐K in magenta and DAPI in blue. White arrowhead points to LWS2‐K in outer segment. **D–D′:** Retina of a Tg(LWS) animal of 8.6‐mm standard length, showing LWS1‐G in green and LWS2‐K in magenta. **D″:** Merge of D and D′ with DAPI in blue. **D″′:** Zoom in on boxed area in D″. **E:** Overview of a pineal gland of a Tg(LWS) larva at 8.6‐mm standard length with LWS2‐K in magenta, LWS1‐G in green, and DAPI in blue. **E′–E″:** Zoom in on boxed area in E. **F:** Reverse transcription polymerase chain reaction results for wild‐type (lane 2 to 5) and TG(LWS) (lane 6 to 9) littermates. cDNA was prepared from 3‐dpf, 5‐dpf, 8.6‐mm standard‐length larvae and adult retinas (ad). Wild‐type amplicons were obtained using *opn1lw1* specific primers. TG(LWS) Polymerase chain reaction products were amplified using mNeonGreen specific primers. Lane 1: marker. Scale bar A = 1 mm. Scale bars B, C, D‐D″, E =100 μm. Scale bars D″′, E′, E″ = 10 μm. Scale bar C′ = 10 μm.

To investigate the temporal and spatial distribution of LWS1‐G and LWS2‐K during Zebrafish retinal development, expression of the fusion proteins was analyzed in both live embryos and fixed retinal sections of WT and Tg(LWS) siblings. LWS2‐K was first detected at 2 dpf in the PRCs of the pineal gland in live Zebrafish embryos (Fig. 2A, arrowhead) and continued to be expressed in these cells until adulthood.

In the PRCs of the retina, LWS2‐K could be easily visualized up to 5 dpf in live embryos treated with 1‐Phenyl‐2‐thiourea (PTU), which prevents the production of melanin (Karlsson et al., 2001). During later stages of development, visualization of tagged red opsins was not possible without sectioning the tissue, since keeping fish in PTU was no longer feasible. To this end, and to obtain a better spatial resolution, we assessed reporter fluorescence in retinal sections.

In the retina, LWS2‐K was first detected at about 3 dpf (Fig. 2B–C′) in the developing OS, which is in good agreement with the expression of the endogenous protein (Mitchell et al., 2015; Takeuchi et al., 2011). The number of LWS2‐K‐positive cells varied between embryos of the same clutch (Fig. 2B,C). This result was confirmed in the progeny of all Tg founders analyzed (n=3), suggesting it is not a “positional effect” of a single transgenic line. A similar phenomenon was previously reported for an independent red opsin transgenic line. In contrast to the broad mRNA expression domain, the fluorescence from this reporter was patchy (Mitchell et al., 2015). This difference between spatial distribution of mRNA and protein could be explained by a translational delay in some cells or might be inherent to the transgene.

To determine the onset of LWS1‐G fluorescence, we used the same imaging parameters as for detection of LWS1‐G in the Tg(LWS) adult retina. These settings were chosen carefully to avoid bleed‐through of the LWS2‐K signal. At these specific imaging parameters, we could not detect any LWS1‐G signal at any stage of early Zebrafish development (up to 5 dpf). However, we cannot exclude the presence of faint LWS1‐G signals, which are below the detection limit and which would be masked at higher excitation powers by bleed‐through of the strong mKate2 signal.

To determine the onset of LWS1‐G fluorescence in the developing retina, we performed a time series of retinal sections. As suggested previously, staging of Zebrafish larvae older than 5 dpf was based on body length and has been described to be a more accurate way to reproducibly stage juvenile Zebrafish (Parichy et al., 2009). LWS1‐G fluorescence was first detected in the retina and pineal gland of juvenile Zebrafish at 8.6 mm standard length (SL), corresponding to roughly 14 dpf (Fig. 2D–E″). LWS1‐G could be detected only in the periphery of the ventral region of the retina. In contrast, LWS2‐K, which was initially visible in cells across the entire retina, became restricted to the central and dorsal regions of the retina at this stage (Fig. 2C–2D″′). The fact that LWS2‐K does not accumulate across the entire retina over time suggests that the opsin fusion protein reflects the endogenous protein dynamics.

## Halfway

According to previous reports, *opn1lw1* transcription starts between 3.5 dpf and 7 dpf (Mitchell et al., 2015; Takechi and Kawamura, 2005). In contrast, we could first detect LWS1‐G fluorescence in Tg(LWS) larvae at 8.6 mm SL (∼14 dpf). This difference between the onset of endogenous mRNA transcription and the first detectable mNeonGreen signal raised the question whether transcription from *lws1‐g* encoded by the transgene was delayed in Tg(LWS). To answer this question, we compared mRNA expression of *opn1lw1* in WT with that of *mNeonGreen* mRNA in Tg(LWS) animals at different developmental stages (3 dpf, 5 dpf, 8.6 mm) by reverse transcription polymerase chain reaction (RT‐PCR). As a positive control, we used RNA from adult retinas known to express *opn1lw1* and *lws1‐g* (Fig. 2F). As a negative control, we used mNeonGreen‐specific primers on RNA isolated from WT adult retinas, which did not result in any amplification product (data not shown). Both *opn1lw1* and *lws1‐g* mRNAs were expressed as early as 3 dpf and were continuously detected throughout all stages analyzed (Fig. 2F). This shows that there is no delay between the onset of endogenous and transgenic mRNA transcription. Rather, there is a delay of about 1.5 weeks between the transcription of *lws1‐g* and its first detectable fluorescent signal. This difference is puzzling and cannot simply be explained by a slow maturation of the fluorophore itself. In fact, we chose mNeonGreen as reporter protein because it is known to fold and mature very quickly (Shaner et al., 2013), which makes it ideal to visualize even very dynamic expression patterns, such as oscillating gene expression of cyclic genes (D.S.'s own observation). Furthermore, LWS1‐G localizes correctly in the PRCs and pineal glands at later stages of development, which indicates that no important cues for transcription and translation were disrupted by our transgenesis approach. We cannot exclude that the fusion of mNeonGreen to the C‐terminus of Opn1lw1 per se has an effect on its translation efficiency. However, this seems rather unlikely. To tackle whether the delayed onset of LWS1‐G fluorescence is also an intrinsic feature (translational control) of red opsins, future studies need to assess the presence of mature Opn1lw1 protein.

### OS Size is Not Significantly Affected in Tg(LWS)

**Figure 3:**
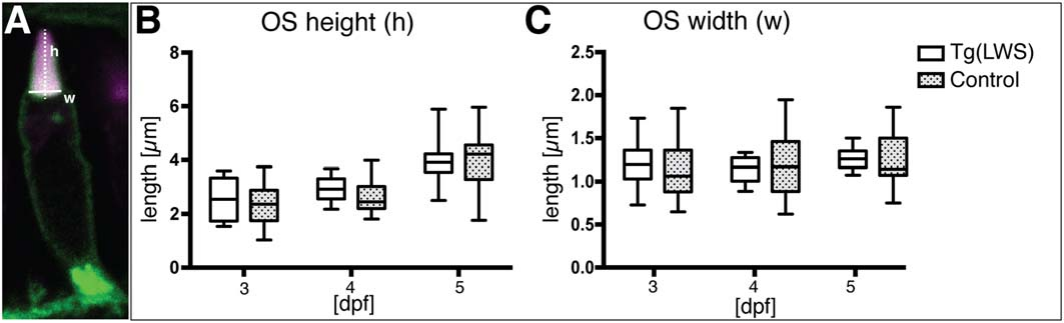
No obvious changes were detected in outer segment size of Tg(LWS) PRCs. **A:** Photoreceptor cell of Tg(LWS)/Tg(Ola.Actb:Hsa.HRAS‐EGFP)^vu119^ at 3 dpf showing mKate2 in magenta and green fluorescent protein at the plasma membrane in green. Outer segment height (h, dotted line) was calculated by measuring the distance from the midpoint at the base of the outer segment to the tip. Outer segment width (w, full line) was calculated by measuring the length of the OS base. **B,C:** Outer segment height (B) and width (C) were measured in both Tg(LWS)/ Tg(Ola.Actb:Hsa.HRAS‐EGFP)^vu119^ (white box) and Tg(Ola.Actb:Hsa.HRAS‐EGFP)^vu119^ stained with red opsin antibody (gray box with dots) at 3 dpf, 4 dpf, and 5 dpf. Measurements are represented in box plots with calculated minimum, 25th percentile, mean, 75th percentile, and maximum. The *x*‐axis shows the age of the embryos in days postfertilization, and the *y*‐axis shows the length measured in μm. For all time points and different transgenic lines, at least three independent samples were used and 20 measurements were carried out. Statistical significance was calculated by a one‐way ANOVA followed by Tukey's multiple comparison test. No statistically significant differences were observed.

Physiological expression levels of opsin proteins are important for correct OS formation. Overexpression of rhodopsin from a transgene in the mouse retina was shown to impact the size of rod OS (Wen et al. 2009; Makino et al., 2012). The transgene Tg(LWS) used here encodes full‐length, presumably active, red opsin fusion proteins, which may result in a gain‐of‐function phenotype. To investigate this, we quantified OS size by measuring OS height and OS width in Tg(LWS) and control PRCs. Clear visualization and measurements of OS parameters in control PRCs using the red opsin antibody were hampered by a low signal. To correctly quantify OS length, the antibody staining for red opsin was performed in a transgenic line, Tg(Ola.Actb:Hsa.HRAS‐EGFP)^vu119^, which allows clear OS visualization by labeling all plasma membranes with GFP. The height of the OS was quantified by measuring the distance from the midpoint of the base to the tip of the OS, and OS width was determined as the length of the OS base (Fig. 3A). No significant difference in OS height and width was observed in PRCs of double transgenics (Tg(LWS)/Tg(Ola.Actb:Hsa.HRAS‐EGFP)^vu119^) compared with single transgenic animals (Tg(Ola.Actb:Hsa.HRAS‐EGFP)^vu119^) stained with red opsin antibody (Fig. 3B,C). This suggests that Tg(LWS) does not trigger significant alterations in OS size.

### Future Potential Uses of Tg(LWS) to Study OS Formation, Maintenance, and Degeneration

The OS of PRCs is a very dynamic structure. Opsins need to be transported from the cytoplasm to the OS, and the OS itself is continuously renewed. We believe that Tg(LWS) could act as tool to study these processes in vivo because we show that the fusion proteins localize to the OS without any obvious signs of accumulation over time, reflecting the protein dynamics of LWS1 and LWS2 independent of each other. Additionally, the transgenic line could be used to directly measure OS volume and growth using image segmentation.

**Figure 4:**
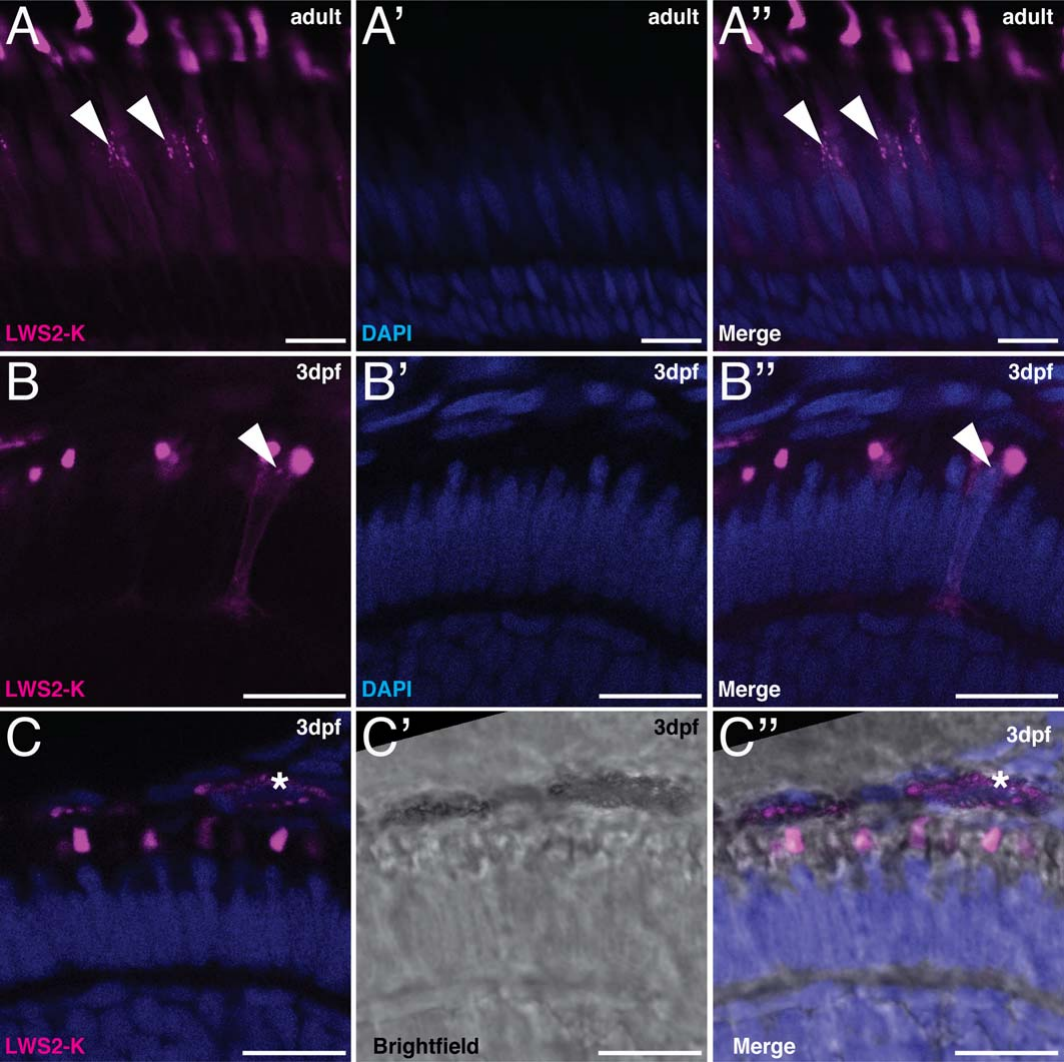
LWS2‐K signals in embryonic and adult retinas. Retinal sections of Tg(LWS) with LWS2‐K in magenta and DAPI in blue. **A–B″:** Vesicle‐like structures are present in the inner segment of LWS2‐K–positive cells. **A–A″:** Retinal sections of adult Tg(LWS). **B–B″:** Retinal sections of Tg(LWS) at 3 dpf. **C–C″:** Retinal sections of Tg(LWS) with brightfield image in gray scale. Asterisks indicate the LWS2‐K–positive signal in the retinal pigment epithelium, which is likely due to the pigment present in these cells. Scale bars A–C″ = 10 μm.

At high excitation powers, the signal emitted by the OS oversaturates the image due to the large amount of protein present in this structure. Interestingly, under these conditions, we could visualize fainter fluorescent structures in the inner segment of PRCs with the same imaging settings (Fig. 4A–B″, arrowhead). This is true for both developing and mature cells. These vesicular structures could be involved in opsin trafficking, which is essential during OS formation, maintenance, and degeneration. However, more detailed studies with vesicle markers, such as Rab proteins, would be needed to further investigate this phenomenon. Live imaging of this line would ensure the required temporal resolution to make direct measurements of this dynamic process, which cannot be achieved using fixed samples.

In the mature retina, parts of the OS are constantly shed and phagocytosed by retinal pigment epithelium (RPE) cells. To determine whether LWS2‐K, produced by PRCs, could be detected in RPE phagosomes, we imaged RPE cells of developing embryos at 3 dpf and 5 dpf. Unfortunately, the analysis was hampered by the autofluorescence of the RPE pigment, which was not entirely blocked by PTU (Fig. 4C–C″). To avoid autofluorescence, Tg(LWS) could be crossed into the *crystal* mutant background (Antinucci and Hindges, 2016), which completely blocks the formation of pigments in the RPE.

## Conclusion

In this study, we generated and characterized a novel Zebrafish transgenic line, Tg(LWS), which mimics the endogenous distribution of Opn1lw1 and Opn1lw2 during retinal development and adulthood. This line provides a tool for various applications, including the analysis of expression, protein localization and trafficking, and opsin switching. Furthermore, this line can now be applied to study in more detail mutants known to affect OS formation, such as cc2d2a and ift88 mutants (Bachmann‐Gagescu et al., 2011; Sukumaran and Perkins, 2009), and OS degeneration (Wasfy et al., 2014; Yin et al., 2011). Finally, this transgenic line can be employed in drug screens to identify novel reagents affecting OS formation, maintenance, and degeneration.

## Experimental Procedures

### Animal Husbandry

Zebrafish were maintained at 28°C under standard conditions in a 14‐hr‐on/10‐hr‐off light/dark cycle. All embryos used were raised in E3 medium in a dark incubator at 28.5°C until 5 dpf. Staging of embryos was done based on age up to 5 dpf, and from 5 dpf onward, Zebrafish were staged based on SL as previously described (Parichy et al., 2009). All animal studies were performed in accordance with European and German animal welfare legislation. Protocols were approved by the Institutional Animal Welfare Officer (Tierschutzbeauftragter), and necessary licenses were obtained from the regional Ethical Commission for Animal Experimentation of Dresden, Germany (Tierversuchskommission, Landesdirektion Sachsen).

### Generation of Transgenic Lines

BAC recombineering was carried out as described in Soroldoni et al., 2014. In short, the BAC CH73‐75L14, spanning the entire region of interest, was identified using Ensembl (https://ensembl.org/) (Yates et al., 2016) and ordered from BACPAC resources (https://bacpacresources.org/). Opn1l1w1 and Opn1l1w2 were tagged sequentially with codon‐optimized versions of mNeonGreen and mKate2 (see Table 1 for primers), respectively.

**Table 1:** Primers used for generation of transgenes

**Tagging primers**	
opn1lw1_F	GCTCTGAGGTGTCCACATCCAAAACAGAAGTGTCTTCTGTGGCTCCTGCAGGAGGAAGCGGAGGAAGC
opn1lw1_R	GTCTCATTTTCATCTTTTTCCCCATGTCTGACTCAGATCTGGTGCACAACCGTCAGTCAGTACCGTTCG
opn1lw2_F	GCTCTGAGGTGTCCACATCCAAAACAGAAGTGTCTTCTGTGGCTCCTGCAGGAGGAAGCGGAGGAAGC
opn1lw2_R	AAGTCCAGTTCTTCCCTCTTGTTCAACAGGAGCTATAAATCACGTAAGACCGTCAGTCAGTACCGTTCG
**Subcloning primers**	
opn1lw1/2_F	TCACTGCTAGAGTGGTTCGTCCGCAGGATGAGGTTACATGAGAACTGTGTCTATAGTGTCACCTAAATC
opn1lw1/2_R	ATTTACTTTTAACCTACAGTCTATGGAACTCACCGTGCTGGTTTCAACCGCCCTATAGTGAGTCGTATTA

In a third recombination step, the modified region of interest was subcloned from the BAC into a plasmid backbone (pShave) containing I‐SceI Meganuclease sites. The resulting construct TgBac(‐2.6opn1lw1:opn1lw1‐mNeonGreen/ opn1lw2‐mKate) comprised the entire endogenous locus and none of the neighboring open‐reading frames up‐/downstream.

Stable transgenic lines were generated as described previously (Soroldoni et al., 2009), using 100 ng/μl of DNA and a bolus size of 100 μm. All injected embryos were raised irrespective of their transient transgene expression levels, which aimed to avoid any visual bias toward strong expressing founders. Transgenic founders were isolated by outcrossing to WT fish (n=10), and their progeny was screened with a fluorescent stereomicroscope (Olympus, SZX16) equipped with appropriate filter sets (AHF, mNeonGreen: F49‐500, F48‐515, F47‐521; mKate2: F49‐560, F48‐585, F47‐630) and a metal‐halide lamp (Excelitas Technologies, X‐cite 120). Five of the seven clutches obtained were mKate2‐positive and showed transgenic offspring at various transmission frequencies.

### Visualization of Endogenous Fluorescence from Tg(LWS) Animals

#### Flat‐mounted retinas

Adult Zebrafish retinas were collected, anesthetized using MESAB (0.2% ethyl‐m‐aminobenzoate methanesulfonate) (Sigma‐Aldrich), and killed by cutting their heads. Retinas were dissected using forceps and fixed in 4% paraformaldehyde (PFA) in phosphate‐buffered saline (PBS) for 2 hr at room temperature. Retinas were then cut, placed on a drop of Vectashield antifade mounting medium (Vector Labs) on top of a glass slide, and flattened using a coverslip.

#### Retinal sections

Zebrafish at 3 dpf, 5 dpf, 4.9 mm SL, 5.9 mm SL, 6.4 mm SL, 7.2 mm SL, 8.6 mm SL, 11 mm SL, and 13 mm SL were collected, anesthetized (see above), and killed by cutting their heads. Up to 13 mm SL, heads were directly fixed in 4% PFA. For adults, eyes were dissected. One eye was used for RNA extraction and the other was fixed in 4% PFA overnight at 4°C. Fixed heads and eyes were washed twice for 10 min in 1X PBS and kept for 1 hr in 5% sucrose in 1X PBS, followed by overnight incubation in 30% Sucrose in 1X PBS at 4°C. Finally, eyes were incubated for 1 hr at room temperature in a 1:1 solution of 30% Sucrose in 1X PBS/ NEG‐50™ (Thermo Fisher Scientific) and mounted in NEG‐50™ and frozen in dry ice. All samples were kept at ‐80°C until sectioning. Zebrafish eyes were cut into 16‐μm sections and left to dry for at least 1 hr at room temperature. After sectioning, all samples were kept at ‐20°C for a maximum of 3 weeks. Before staining and imaging, retinal sections were dried for at least 1 hr at room temperature followed by rehydration in 1X PBS for 30 min. For nuclear staining, sections were incubated for 1 min in DAPI (Thermo Fischer Scientific) diluted 1:10000 in PBS at room temperature. Finally, sections were washed three times for 5 min at room temperature and mounted in Vectashield antifade mounting medium (Vector Labs).

#### Live embryos

2‐dpf Tg(LWS) Zebrafish were anesthetized using MESAB (see above) and mounted in 1% low‐melting agarose (Sigma‐Aldrich).

### Immunohistochemistry

Immunostainings of Zebrafish retina were performed on adult retinal sections as described previously (Thummel et al., 2008). 16‐μm sections were incubated with a rabbit polyclonal antibody raised against the N‐terminus of Zebrafish red opsin (Vihtelic et al., 1999) in a 1:50 dilution overnight at room temperature. Alexa Fluor 488‐ and Alexa Fluor 564‐conjugated anti‐rabbit secondary antibodies (diluted 1:500) were incubated overnight at 4°C. All samples were mounted in Vectashield antifade medium (Vector Labs).

### Imaging

All samples were imaged with a ZEISS multiphoton laser‐scanning upright microscope (https://www.biodip.de/wiki/MZ1_-_Zeiss_2photon_upright) using a Zeiss Plan Neofluar 20x NA 0.8, or Zeiss Plan Neofluar 63x NA 0.8 objective. All images were acquired using ZEN 2011 software (black edition). All images obtained from ZEN software were analyzed using Fiji (Schindelin et al., 2012).

### RT‐PCR

Zebrafish retinas were dissected as described above and homogenized in 1 ml of TRIzol (Invitrogen, Carlsbad, CA). Total RNA was extracted following the manufacturer's protocol. Prior to cDNA synthesis, RNA was treated with DNase (Invitrogen) for 30 min at 37°C. The reaction was stopped using DNase stopping solution (Invitrogen), and the entire solution was used for cDNA synthesis. 0.5 μg of total RNA was used for cDNA synthesis using the SuperScript II Reverse Transcriptase (Invitrogen).

PCR amplification of cDNA was done using Template cDNA (20–50 ng), forward primer (1 μl [stock 10 μM]), reverse primer (1 μl [stock 10 μM]), dNTP (5 μl stock 2 mM), 5X HQ buffer (10 μl), and Phusion DNA polymerase (0.5 μl) (Invitrogen) with the following cycling conditions—95°C for 3 min followed by 35 cycles of the following temperature regime: 98°C for 30 sec, 55°C for 30 sec, 72°C for 1 min, and a final elongation at 72°C for 10 min. (See Table 2.)

**Table 2:** Primers used for RT‐PCR

RT‐PCR primers	
opn1lw1_F	TGCATCTCGACAACTCTGCT
opn1lw1_R	GGCAGGCATCTACCTATCACT
*mKate2*_F	TCAAACAGTCCTTCCCCGAG
*mKate2*_R	CGTAGTACACTCCGGGCATT
*mNeonGreen_F*	ACCAGTACCTGCCTTACCCT
*mNeonGreen_R*	GCAGCCATAGGCTTAGCGA

### Quantification of Outer Segments

Both OS height and width were measured using Fiji (Schindelin et al., 2012). Graphs and statistical analyses were done using GraphPad Prism 6. The statistical significance was calculated by a one‐way ANOVA followed by a Tukey's multiple comparison test.

## Supporting information

Additional supporting information may be found in the online version of this article.

Supporting Information
