# Supplementary material for: A novel transgenic zebrafish line for red opsin expression in outer segments of photoreceptor cells
Source: Dev Dyn. 2018 Apr 23;247(7):951–9. doi: 10.1002/dvdy.24631 (PMC6099204; doi:10.1002/dvdy.24631)
Supplement: Supplementary file 1 — Supporting Information [file DVDY-247-951-s001.pdf]

Developmental Dynamics

A novel transgenic zebrafish line for red opsin expression in outer segments of photoreceptor cells

Journal:	*Developmental Dynamics*
Manuscript ID	DVDY-18-0008.R1
Wiley - Manuscript type:	Patterns & Phenotypes
Date Submitted by the Author:	16-Mar-2018
Complete List of Authors:	Crespo, Cátia; Max Planck Institute of Molecular Cell Biology and Genetics<br>Soroldoni, Daniele; École Polytecnique Fédérale de Lausanne , UPOATES, Station 19<br>Knust, Elisabeth; Max Planck Institute of Molecular Cell Biology and Genetics,
Keywords:	retina, cone cells, development, LWS1, LWS2

SCHOLARONE™
Manuscripts

A novel transgenic zebrafish line for red opsin expression in outer segments of photoreceptor cells

Cátia Crespo, Daniele Soroldoni[#]* and Elisabeth Knust*

Max-Planck-Institute of Molecular Cell Biology and Genetics,
Pfotenhauerstrasse 108
01307-Dresden, Germany

For Peer Review

[#] École Polytecnique Fédérale de Lausanne
UPOATES
Station 19
CH-1015 Lausanne
Switzerland

* Corresponding author
Tel: +49-351-210-1300
Fax: +49-351-210-1309
e-mail: knust@mpi-cbg.de
e-mail: daniele.soroldoni@epfl.ch

Running title: Red-opsin reporter

Keywords: development, retina, cone cells, LWS1, LWS2

Abstracts

Background: Opsins are a group of light sensitive proteins present in photoreceptor cells, which convert the energy of photons into electrochemical signals, thus allowing vision. Given their relevance, we aimed to visualise the two red opsins at subcellular scale in photoreceptor cells.

Results: We generated novel zebrafish BAC transgenic lines, which express fluorescently-tagged, full-length Opsin 1 long-wave-sensitive 1 and full-length Opsin 1 long-wave-sensitive 2 under the control of their endogenous promotors. Both fusion proteins are localised in the outer segments of photoreceptor cells. During development, Opn1lw2-mKate2 is detected from the initial formation of outer segments onwards. In contrast, Opn1lw1-mNeonGreen is first detected in juvenile zebrafish at about 2 weeks post fertilisation and both opsins continue to be expressed throughout adulthood. Importantly, the presence of the transgene did not significantly alter the size of outer segments.

Conclusions: We have generated multiple transgenes that mimic the endogenous expression pattern of Opn1lw1 and Opn1lw2 in the developing and adult retina. In contrast to existing lines, our transgene design allows to follow protein localisation. Hence, we expect that these lines could act as useful real-time reporters to directly measure phenomena in retinal development and disease models.

For Peer Review

Introduction

Photoreceptor cells (PRCs) are specialised neurons, which, in vertebrates, are classified into rods, specialised for dim light vision, and cones (Mustafi et al., 2009), responsible for bright light and colour vision (Carter-Dawson and Lavail, 1979; Kawamura and Tachibanaki, 2008). All PRCs are characterised by a pronounced apico-basal polarity, with the apical domain subdivided into the inner segment (IS) and the outer segment (OS). The OS is a modified primary cilium essential for phototransduction. The OS of developing and mature PRCs is characterised by a tremendously expanded apical membrane in order to accommodate the huge amount of visual pigment [reviewed in (May-Simera et al., 2017)]. The visual pigment consists of a transmembrane protein, opsin, linked to a chromophore, which is responsible for absorption of photons of light (Allison et al., 2004; Enright et al., 2015; Samardzija et al., 2009; Saszik and Bilotta, 1999; Suliman and Novales Flamarique, 2014; Yokoyama, 2000). Correct opsin localisation in the OS requires active transport of the protein from the cell body into the OS and is essential for PRC function and survival [reviewed in (Bales and Gross, 2016; Hollingsworth and Gross, 2012)].

In zebrafish, opsins have been classified into five different groups according to the wavelength they absorb. Due to genome duplications, 10 different opsin genes are encoded in the zebrafish genome: two rhodopsin genes (Morrow et al., 2017), one blue and one ultraviolet opsin gene, four different green opsin genes and two red opsin genes. Opsin genes are expressed in different PRC subtypes. Rhodopsin is found in rod PRC. Cones can be classified into four different subtypes according to the opsin they express, namely the ultraviolet, blue, red and green sensitive cones (Branchek and Bremiller, 1984; Chinen et al., 2002; Minamoto and Shimizu, 2005).

In zebrafish, the two red opsin genes (*opn1lw1* and *opn1lw2*) are found in a tandem array (head to tail) on the same chromosome and it was shown that they share their regulatory elements (Tsujimura et al., 2010). This locus was used to study the transcriptional regulation of tandemly-replicated opsins to demonstrate that expression can switch from one to the other paralog, which was referred to as opsin switching (Mitchell et al., 2015). Furthermore, red opsins are expressed in one of the most abundant cone PRCs.

For Peer Review

Several zebrafish reporter lines have been generated in the past, which allow to discriminate the different PRC subtypes. In most cases, endogenous promoters drive the expression of fluorescent proteins, which replace or disrupt the coding sequence of the respective gene of interest (DuVal et al., 2014; Fadool, 2003; Fang et al., 2017, 2013; Fraser et al., 2013; Hagerman et al., 2016; Hamaoka et al., 2002; Kennedy et al., 2007, 2001; Li et al., 2012; Luo et al., 2004; Ogawa et al., 2015; Raymond et al., 2014; Suzuki et al., 2013; Takechi et al., 2008, 2003, Tsujimura et al., 2010, 2007; Yu et al., 2007; Zou et al., 2010). Therefore, these transgenic lines can be considered transcriptional reporters, which can be used to trace the onset of transcription and track cells that express the transgene, but fail to report the dynamics of (protein) expression due to the prolonged stability of the fluorescent protein. In addition, proteins expressed from these reporter lines typically lack functional opsin domains, which are required for proper protein (subcellular) localisation and stability (Mitchell et al., 2015). Thus, fluorescent proteins are restricted to the cytoplasm of PRCs and are not actively transported to the OS.

To visualise the OS of rod PRCs, a transgenic zebrafish line has been established, which encodes a *Xenopus* Rhodopsin-GFP fusion protein, expressed under the control of the *Xenopus* rhodopsin promoter (Perkins et al., 2002; Tam et al., 2000). This line overcomes the technical limitations mentioned above, but it can only be used to study rods (Perkins et al., 2002), the best studied PRCs to-date. Cones, on the other hand, which are essential for colour vison, have received less attention so far. The zebrafish retina contains predominantly cones, and one of the most abundant cones are those that express the red opsins. This makes *opn1lw1* and *opn1lw2* ideal candidates to study cone OS maturation and maintenance (Raymond et al., 1995).

In this study, we generated a new BAC transgene that comprises the endogenous loci of the two red opsins, *opn1lw1* and *opn1lw2*. The transgene was designed to express opsin fusion proteins, which can be transported to the OS. This line now allows to distinguish the expression and localisation of both red opsins during maturation and maintenance of the OS at high spatial and temporal resolution. Furthermore, this transgenic line has the potential to be used as real-time reporter to analyse OS formation and degeneration, opsin trafficking and switching.

For Peer Review

Results and discussion

Generation of a transgenic line expressing the two red opsins

To study the OS of cones, we decided to focus our attention on red sensitive cones, which are amongst the most abundant in the zebrafish retina. To this end, we employed BAC recombineering to embed fluorescent reporter cassettes into the red opsin loci, *opn1lw1* and *opn1lw2,* which are arranged head to tail on the same chromosome (Fig.1 A). The BAC contained all regulatory regions, which are shared by the two loci (Allison et al. 2010; Salbreux et al. 2012; Chinen et al. 2003; Branchek & Bremiller 1984; Minamoto & Shimizu 2005). Unlike previously published transgenes, in which the coding regions are disrupted (Mitchell et al., 2015; Tsujimura et al., 2010), we replaced the stop codons of *opn1lw1* and *opn1lw2* with mNeonGreen and mKate2, respectively, which resulted in two intact C-terminal fusion proteins. This approach aimed to exploit all regulatory cues, harboured in the endogenous locus, which are required to mimic reporter transcription, translation and stability. The latter is essential for the 'temporal resolution' of transgene expression and needs to be considered if a transgene should be used as a real-time reporter.

To avoid overexpression of untagged open reading frames present on the BAC and to increase the transgenesis frequency, which is typically very low for large transgene constructs, we introduced a second recombination step to subclone the modified region of interest into a plasmid backbone containing I-SceI Meganuclease sites. This 'shaved' BAC (approx. 12 kb in size) included the 2.6 kb upstream region of *opn1lw1* (Fig. 1A), which was shown to be required and sufficient to drive *opn1lw1* and *opn2lw2* transcription (Tsujimura et al., 2010).

The resulting construct was co-injected with I-SceI Meganuclease into zebrafish embryos. All injected embryos were raised until adulthood and crossed with wildtype (WT) animals. Their progeny was screened at 5 days-post-fertilisation (dpf) with a fluorescent stereomicroscope, and five out of the seven screened clutches showed progeny that was mKate2 positive. At this stage, Opn1lw2-mKate2 (LWS2-K) was visible in live embryos in the retina and the pineal gland, but Opn1lw1-mNeonGreen (LWS1-G) could not be detected. We compared the progenies of five independent founders and did not find obvious differences in the onset of LWS2-K fluorescence, intensity or spatial distribution of the fusion protein. Thus, all further studies were performed with the progeny of one transgenic

For Peer Review

founder, designated as TgBac(-2.6opn1lw1:opn1lw1-mNeonGreen/opn1lw2-mKate) (cbg9Tg at ZFIN, https://zfin.org/ZDB-ALT-180320-10), which we will refer to as Tg(LWS) from now on. In order to test the stability of the transgene insertion, transgenic animals were continuously outcrossed with WT fish until the F4 generation was obtained. LWS2-K expression was detected throughout all generations, indicating that the transgene was stably inserted in the zebrafish genome.

Localisation of transgene-expressed protein in outer segments

As stated above, LWS2-K could be detected in the early stages of the zebrafish retina, indicating the presence and activity of the transgene. However, LWS1-G could not be detected. To investigate if LWS1-G is active at all and to see if LWS2-K is maintained, we examined retinas of adult transgenic fish. Flat-mounted adult retina clearly showed the activity of both transgenes (Fig 1B-B''). To further study the subcellular localisation of both fusion proteins, we performed cryo-sections and imaged the fluorescence of fusion proteins without any further amplification of the signal in fixed tissues. In Tg(LWS) adult retinas, both LWS1-G and LWS2-K were restricted to the PRC layer, distal to the outer nuclear layer (Fig. 1C-C''). In accordance with previous reports (Takechi and Kawamura, 2005; Tsujimura et al., 2010), the number of LWS1-G positive cells was highest in the ventral-most region, with a few positive cells on the dorsal side, while LWS2-K was restricted to the central region in the adult retina (Fig. 1B-C''). Some LWS1-G positive cells could be detected in the central retina and some LWS2-K were found in the peripheral retina (Fig. 1B-C'').

To further confirm the subcellular localisation of the fusion proteins in the OS, we conducted immunohistochemistry with a polyclonal antibody raised against zebrafish red opsin. This antibody was shown to recognise red opsin proteins in the retina and a protein of the predicted size in western blots (Tsujimura et al., 2010; Vihtelic et al., 1999). Retinal sections of adult WT and Tg(LWS) siblings were prepared and processed in parallel (on the same slide) to control the immunohistochemistry protocol. In Tg(LWS) we found that the fluorescence of LWS2-K and red opsin antibody signal largely overlaps in the OS of PRCs (Fig. 1D-D''). At first, this correlation was not surprising since the polyclonal antibody could recognise the endogenous and transgenic protein. However, we could not detect a significantly stronger signal when we compared retina sections of WT and TG littermates stained with the antibody on the same slide. This raises the interesting possibility that the amount of opsin present in the OS is regulated. However, not all antibody-positive cells

For Peer Review

showed reporter fluorescence. This could have various reasons. Fixation of the sample could affect protein structure, which can impair fluorescence intensity (Kusser & Randall 2003). Alternatively, transgene expression could be silenced in some LWS cones. The discrepancy between the antibody staining and the fluorescence could be resolved by further experiments using monoclonal antibodies raised against red opsins.

LWS1-G and LWS2-K are differentially expressed during retinal development

To investigate the temporal and spatial distribution of LWS1-G and LWS2-K during zebrafish retinal development, expression of the fusion proteins was analysed in both live embryos and fixed retinal sections of WT and Tg(LWS) siblings. LWS2-K was first detected at 2 dpf in the PRCs of the pineal gland in live zebrafish embryos (Fig. 2A, arrowhead) and continued to be expressed in these cells until adulthood.

In the PRCs of the retina LWS2-K could be easily visualised up to 5 dpf in live embryos treated with 1-Phenyl-2-thiourea (PTU), which prevents the production of melanin (Westerfield, 2007). During later stages of development, visualisation of tagged red opsins was not possible without sectioning the tissue, since keeping fish in PTU is no longer feasible. To this end and to obtain a better spatial resolution, we assessed reporter fluorescence in retinal sections.

In the retina, LWS2-K was first detected at about 3 dpf (Fig. 2B-C') in the developing OS, which is in good agreement with the expression of the endogenous protein (Mitchell et al., 2015; Takeuchi et al., 2011). The number of LWS2-K positive cells varied between embryos of the same clutch (Fig.2 B-C). This result was confirmed in the progeny of all Tg founders analysed (N = 3), suggesting it is not a 'positional effect' of a single transgenic line. A similar phenomenon was previously reported for an independent red opsin transgenic line. In contrast to the broad mRNA expression domain, the fluorescence from this reporter was patchy (Mitchell et al., 2015). This difference between spatial distribution of mRNA and protein could be explained by a translational delay in some cells or might be inherent to the transgene.

To determine the onset of LWS1-G fluorescence, we used the same imaging parameters as for detection of LWS1-G in the Tg(LWS) adult retina. These settings were chosen carefully to avoid bleed-through of the LWS2-K signal. At these specific imaging parameters, we could not detect any LWS1-G signal at any stage of early zebrafish development (up to 5 dpf). However, we cannot exclude the presence of faint LWS1-G

For Peer Review

signals which are below the detection limit and which would be masked at higher excitation powers by bleed-through of the strong mKate2 signal.

In order to determine the onset of LWS1-G fluorescence in the developing retina, we performed a time series of retinal sections. As suggested previously, staging of zebrafish larvae older than 5 dpf was based on body length, has been described to be a more accurate way to reproducibly stage juvenile zebrafish (Parichy et al., 2009). LWS1-G fluorescence was first detected in the retina and pineal gland of juvenile zebrafish at 8.6 mm standard length (SL), corresponding to roughly 14 dpf (Fig. 2D-E''). LWS1-G could only be detected in the periphery of the ventral region of the retina. In contrast, LWS2-K, which was initially visible in cells across the entire retina, became restricted to the central and dorsal regions of the retina at this stage (Fig 2C-2D'''). The fact that LWS2-K does not accumulate across the entire retina over time suggests that the opsin fusion protein reflects the endogenous proteins dynamics.

According to previous reports *opn1lw1* transcriptions starts between 3.5 dpf and 7 dpf (Mitchell et al., 2015; Takechi and Kawamura, 2005). In contrast, we could first detect LWS1-G fluorescence in Tg(LWS) larvae at 8.6 mm SL (~14 dpf). This difference between the onset of endogenous mRNA transcription and the first detectable mNeonGreen signal raised the question whether transcription from *lws1-g* encoded by the transgene was delayed in Tg(LWS). To answer this question, we compared mRNA expression of *opn1lw1* in WT with that of *mNeonGreen* mRNA in Tg(LWS) animals at different developmental stages (3 dpf, 5 dpf, 8.6 mm) by RT-PCR. As a positive control, we used RNA from adult retinas known to express *opn1lw1* and *lws1-g* (Fig. 2F). As a negative control, we used mNeonGreen specific primers on RNA isolated from WT adult retinas, which did not result in any amplification product (data not shown). Both *opn1lw1* and *lws1-g* mRNAs were expressed as early as 3 dpf and were continuously detected throughout all stages analysed (Fig. 2F). This shows that there is no delay between the onset of endogenous and transgenic mRNA transcription. Rather, there is a delay of about 1.5 weeks between the transcription of *lws1-g* and its first detectable fluorescent signal. This difference is puzzling and cannot simply be explained by a slow maturation of the fluorophore itself. In fact, we chose mNeonGreen as reporter protein because it is known to fold and mature very quickly (Shaner et al., 2013), which makes it ideal to visualize even very dynamic expression patterns, such as oscillating gene expression of cyclic genes (DS own observation). Furthermore, LWS1-G localises correctly in the PRCs and pineal glands at later stages of development, which indicates that no important cues for transcription and translation were disrupted by our transgenesis

For Peer Review

approach. We cannot exclude that the fusion of mNeonGreen to the C-terminus of Opn1lw1 *per se* has an effect on its translation efficiency. However, this seems rather unlikely. To tackle whether the delayed onset of LWS1-G fluorescence is also an intrinsic feature (translational control) of red opsins, future studies need to assess the presence of mature Opn1lw1 protein.

OS size is not significantly affected in Tg(LWS)

Physiological expression levels of opsin proteins are important for correct OS formation. Overexpression of rhodopsin from a transgene in the mouse retina was shown to impact the size of rod OS (Wen et al. 2009). The transgene Tg(LWS) used here encodes full length, presumably active, red opsin fusion proteins, which may result in a gain-of-function phenotype. To investigate this, we quantified OS size by measuring OS height (h) and OS width (w) in Tg(LWS) and control PRCs. Clear visualisation and measurements of OS parameters in control PRCs using the red opsin antibody was hampered by a low signal. To correctly quantify OS length, the antibody staining for red opsin was performed in a transgenic line, Tg(Ola.Actb:Hsa.HRAS-EGFP)^{vu119}, which allows clear OS visualisation by labelling all plasma membranes with GFP. The height of OSs was quantified by measuring the distance from the mid-point of the base to the tip of the OS, and OS width was determined as the length of the OS base (Fig. 3A). No significant difference in OS height and width was observed in PRCs of double transgenics (Tg(LWS)/ Tg(Ola.Actb:Hsa.HRAS-EGFP)^{vu119}) compared with single transgenic animals (Tg(Ola.Actb:Hsa.HRAS-EGFP)^{vu119}) stained with red opsin antibody (Fig. 3B-C). This suggests that Tg(LWS) does not trigger significant alterations in OS size.

Future potential uses of Tg(LWS) to study OS formation, maintenance and degeneration

The OS of PRCs is a very dynamic structure. Opsins need to be transported from the cytoplasm to the OS and the OS itself is continuously renewed. We believe that Tg(LWS) could act as tool to study these processes in vivo because we show that the fusion proteins localise to the OS without any obvious signs of accumulation over time, reflecting the protein dynamics of LWS1 and LWS2 independently of each other. Additionally, the transgenic line could be used to directly measure OS volume and growth using image segmentation.

For Peer Review

At high excitation powers the signal emitted by the OS oversaturates the image due to the large amount of protein present in this structure. Interestingly, under these conditions, we could visualise fainter fluorescent structures in the inner segment of PRCs with the same imaging settings (Fig. 4A-B'', arrowhead). This is true for both developing and mature cells. These vesicular structures could be involved in opsin trafficking, which is essential during OS formation, maintenance and degeneration. However, more detailed studies with vesicle markers, such as Rab proteins, would be needed to further investigate this phenomenon. Live imaging of this line would ensure the required temporal resolution to make direct measurements of this dynamic process, which cannot be achieved using fixed samples.

In the mature retina, parts of the OS are constantly shed and phagocytosed by RPE cells. To determine whether LWS2-K, produced by PRCs, could be detected in RPE phagosomes, we imaged RPE cells of developing embryos at 3 and 5 dpf. Unfortunately, the analysis was hampered by the autofluorescence of the RPE pigment, which was not entirely blocked by PTU (Fig. 4C-C''). In order to avoid autofluorescence, Tg(LWS) could be crossed into the *crystal* mutant background (Antinucci and Hindges, 2016), which completely blocks the formation of pigments in the RPE.

For Peer Review

Conclusion

In this study, we generated and characterised a novel zebrafish transgenic line, Tg(LWS), which mimics the endogenous distribution of Opn1lw1 and Opn1lw2 during retinal development and adulthood. This line provides a tool for various applications, including the analysis of expression, protein localisation and trafficking and opsin switching. Furthermore, this line can now be applied to study in more detail mutants known to affect OS formation, such as *cc2d2a* and *ift88* mutants (Bachmann-Gagescu et al., 2011; Sukumaran and Perkins, 2009) and OS degeneration (Wasfy et al., 2014; Yin et al., 2011). Finally, this transgenic line can be employed in drug screens to identify novel reagents affecting OS formation, maintenance and degeneration.

Experimental Procedures

Animal Husbandry

Zebrafish were maintained at 28°C under standard conditions in a 14-hr on/10-hr off light/dark cycle. All embryos used were raised in E3 medium in a dark incubator at 28,5°C

until 5 dpf. Staging of embryos was done based on age up to 5 dpf and from 5 dpf onwards, zebrafish were staged based on standard length as previously described (Parichy et al., 2009). All animal studies were performed in accordance with European and German animal welfare legislation. Protocols were approved by the Institutional Animal Welfare Officer (Tierschutzbeauftragter), and necessary licenses were obtained from the regional Ethical Commission for Animal Experimentation of Dresden, Germany (Tierversuchskommission, Landesdirektion Sachsen).

Generation of Transgenic lines

BAC recombineering was carried out as described in Soroldoni et al 2014. In short, the BAC CH73-75L14, spanning the entire region of interest, was identified using Ensembl (https://ensembl.org/) (Yates et al., 2016) and ordered from BACPAC resources (https://bacpacresources.org/). Opn1l1w1 and Opn1l1w2 were tagged sequentially with codon optimised versions of mNeonGreen and mKate2 (see table for primers), respectively.

**Tagging primers**	
opn1lw1_F	GCTCTGAGGTGTCCACATCCAAAACAGAAGTGTCTTCTGTGGCTCCTGCAGGAGGAA GCGGAGGAAGC
opn1lw1_ R	GTCTCATTTTCATCTTTTTCCCCATGTCTGACTCAGATCTGGTGCACAACCGTCAGTCA GTACCGTTCG
opn1lw2_F	GCTCTGAGGTGTCCACATCCAAAACAGAAGTGTCTTCTGTGGCTCCTGCAGGAGGAA GCGGAGGAAGC
opn1lw2_ R	AAGTCCAGTTCTTCCCTCTTGTTCAACAGGAGCTATAAATCACGTAAGACCGTCAGTC AGTACCGTTCG
**Subcloning primers**	
opn1lw1/2 _F	TCACTGCTAGAGTGGTTCGTCCGCAGGATGAGGTTACATGAGAACTGTGTCTATAGTG TCACCTAAATC
opn1lw1/2 _R	ATTTACTTTTAACCTACAGTCTATGGAACTCACCGTGCTGGTTTCAACCGCCCTATAG TGAGTCGTATTA

In a third recombination step the modified region of interest was subcloned from the BAC into a plasmid backbone (pShave) containing I-SceI Meganuclease sites. The resulting construct TgBac(-2.6opn1lw1:opn1lw1-mNeonGreen/ opn1lw2-mKate) comprised the entire endogenous locus and none of the neighbouring open reading frames up/downstream.

Stable transgenic lines were generated as described previously (Soroldoni et al., 2009), using 100 ng/µl of DNA and a bolus size of 100 µm. All injected embryos were raised

irrespective of their transient transgene expression levels, which aimed to avoid any visual bias towards strong expressing founders. Transgenic founders were isolated by outcrossing to WT fish (n = 10) and their progeny was screened with a fluorescent stereomicroscope (Olympus, SZX-16) equipped with appropriate filter sets (AHF, mNeonGreen: F49-500, F48-515, F47-521; mKate2: F49-560, F48-585, F47-630) and a metal halide lamp (Excelitas Technologies, X-cite 120). Five of the seven clutches obtained were mKate2 positive and showed transgenic offspring at various transmission frequencies.

Visualization of endogenous fluorescence from Tg(LWS) animals

Flat-mounted retinas: Adult zebrafish retinas were collected, anesthetized using MESAB (0.2% ethyl-m-aminobenzoate methanesulfonate - Sigma Aldrich) and killed by cutting their heads. Retinas were dissected using forceps and fixed in 4% paraformaldehyde (PFA, in PBS) for two hours at room temperature. Retinas were then cut, placed on a drop of Vectashield antifade mounting medium (Vectorlabs) on top of a glass slide and flattened using a cover slip.

Retinal sections: Zebrafish at 3 dpf, 5 dpf, 4.9 mm SL, 5.9 mm SL, 6.4 mm SL, 7.2 mm SL, 8.6 mm SL, 11 mm SL and 13 mm SL were collected, anesthetized (see above) and killed by cutting their heads. Up to 13 mm SL, heads were directly fixed in 4% PFA. For adults, eyes were dissected. One eye was used for RNA extraction and the other one was fixed in 4% PFA overnight at 4°C. Fixed heads and eyes were washed twice for ten minutes in 1X PBS and kept for one hour in 5% sucrose in 1X PBS, followed by overnight incubation in 30% Sucrose in 1X PBS at 4°C. Finally, eyes were incubated for one hour at room temperature in a 1:1 solution of 30% Sucrose in 1X PBS/ NEG-50™ (Thermo Fisher Scientific) and mounted in NEG-50™ and frozen in dry ice. All samples were kept at -80°C until sectioning. Zebrafish eyes were cut into 16 µm sections and left to dry for at least one hour at room temperature. After sectioning, all samples were kept at -20°C for a maximum of three weeks. Before staining and imaging, retinal sections were dried for at least one hour at room temperature followed by rehydration in 1X PBS for 30 minutes. For nuclear staining, sections were incubated for ten minutes in DAPI (Thermo Fischer Scientific) diluted 1:10000 in PBS at room temperature. Finally, sections were washed three times for five minutes at room temperature and mounted in Vectashield antifade mounting medium (Vectorlabs).

Live embryos: 2 dpf Tg(LWS) zebrafish were anesthetized using MESAB (see above) and mounted in 1% low melting agarose (Sigma Aldrich).

For Peer Review

Immunohistochemistry

Immunostainings of zebrafish retina were performed on adult retinal sections as described previously (Thummel et al., 2008). 16 µm sections were incubated with a rabbit polyclonal antibody raised against the N-terminus of zebrafish red opsin (Vihtelic et al., 1999) in a 1:50 dilution overnight at room temperature. Alexa Fluor 488- and Alexa Fluor 564-conjugated anti-rabbit secondary antibodies (diluted 1:500) were incubated overnight at 4°C. All samples were mounted in Vectashield antifade medium (Vectorlabs).

Imaging

All samples were imaged using a ZEISS multiphoton laser scanning upright microscope (https://www.biodip.de/wiki/MZ1_-_Zeiss_2photon_upright), using a Zeiss Plan-Neofluar 20x NA 0.8 or Zeiss Plan-Neofluar 63x NA 0.8 objective. All images were acquired using ZEN 2011 software (black edition). All images obtained from ZEN software were analysed using Fiji (Schindelin et al., 2012).

RT-PCR

Zebrafish retinas were dissected as described above and homogenised in one ml of Trizol (Invitrogen, Carlsbad, CA). Total RNA was extracted following the manufacturer's protocol. Prior to cDNA synthesis, RNA was treated with DNase (Invitrogen) for 30 minutes at 37°C. The reaction was stopped using DNase stopping solution (Invitrogen), and the entire solution was used for cDNA synthesis. 0.5 µg of total RNA was used for cDNA synthesis using the SuperScript II reverse transcriptase (Invitrogen).

PCR amplification of cDNA was done using Template cDNA (20-50ng), forward primer (1µl (stock 10µM)), reverse primer (1µl (stock 10µM)), dNTP (5µl-stock 2mM), 5X HQ buffer (10µl) and Phusion DNA polymerase (0.5µl) - Invitrogen) with the following cycling conditions: 95°C for 3 min, followed by 35 cycles of the following temperature regime: 98°C for 30 s, 55°C for 30 s, 72°C for 1 min, with a final elongation at 72°C for 10 min.

**RT-PCR primers**	
opn1lw1_F	TGCATCTCGACAACTCTGCT
opn1lw1_R	GGCAGGCATCTACCTATCACT
mKate2_F	TCAAACAGTCCTTCCCCGAG
mKate2_R	CGTAGTACACTCCGGGCATT
mNeonGreen_F	ACCAGTACCTGCCTTACCCT

mNeonGreen_R	GCAGCCATAGGCTTAGCGA

Quantification of outer segments

Both OS height and width were measured using Fiji (Schindelin et al., 2012). Graphs and statistical analyses were done using GraphPad Prism6. The statistical significance was calculated by a one-way ANOVA followed by a Tukey's multiple comparison test.

Acknowledgments

We would like to thank Dr. Hyde for sharing with us the antibody against red opsin, the MPI-CBG fish facility for helping maintaining our stocks and the MPI-CBG light microscopy facility for guidance. This work was supported by the Max Planck Society. C.C. was a member of the International Max Planck Research School for Cell, Developmental and Systems Biology and a doctoral student at Technische Universität Dresden.

For Peer Review

Bibliography

Allison WT, Barthel LK, Skebo KM, Takechi M, Kawamura S, Raymond PA. 2010. Ontogeny of cone photoreceptor mosaics in zebrafish. J Comp Neurol 518:4182–4195.

Allison WT, Haimberger TI, Hawryshyn CW, Temple SE. 2004. Visual pigment composition in zebrafish: Evidence for a rhodopsin-porphyrop sin interchange system. Vis Neurosci 22:249.

Antinucci P, Hindges R. 2016. A crystal -clear zebrafish for in vivo imaging. Sci Rep 6:29490.

Bachmann-Gagescu R, Phelps IG, Stearns G, Link BA, Brockerhoff SE, Moens CB, Doherty D. 2011. The ciliopathy gene cc2d2a controls zebrafish photoreceptor outer segment development through a role in Rab8-dependent vesicle trafficking. Hum Mol Genet 20:4041–4055.

Bales KL, Gross AK. 2016. Aberrant protein trafficking in retinal degenerations: The initial phase of retinal remodeling 150:71–80.

Branchek T, Bremiller R. 1984. The development of photoreceptors in the zebrafish,Brachydanio rerio. I. Structure. J Comp Neurol 224:107–115.

Carter-Dawson LD, Lavail MM. 1979. Rods and cones in the mouse retina. I. Structural analysis using light and electron microscopy. J Comp Neurol 188:245–262.

Chinen A, Chinen A, Hamaoka T, Hamaoka T, Yamada Y, Yamada Y, Kawamura S,

Kawamura S. 2002. Gene Duplication and Spectral Diversi cation of Cone Visual Pigments of Zebrafish. Genetics 675:663–675.

DuVal MG, Oel AP, Allison WT. 2014. gdf6a Is Required for Cone Photoreceptor Subtype Differentiation and for the Actions of tbx2b in Determining Rod Versus Cone Photoreceptor Fate. PLoS One 9:e92991.

Enright JM, Toomey MB, Sato SY, Temple SE, Allen JR, Fujiwara R, Kramlinger VM, Nagy LD, Johnson KM, Xiao Y, How MJ, Johnson SL, Roberts NW, Kefalov VJ, Peter Guengerich F, Corbo JC. 2015. Cyp27c1 red-shifts the spectral sensitivity of photoreceptors by converting Vitamin A1into A2. Curr Biol 25:3048–3057.

Fadool JM. 2003. Development of a rod photoreceptor mosaic revealed in transgenic zebrafish. Dev Biol 258:277–290.

Fang W, Bonaffini S, Zou J, Wang X, Zhang C, Tsujimura T, Kawamura S, Wei X. 2013. Characterization of transgenic zebrafish lines that express GFP in the retina, pineal gland, olfactory bulb, hatching gland, and optic tectum. Gene Expr Patterns 13:150–159.

Fang W, Guo C, Wei X. 2017. *Rainbow* Enhancers Regulate Restrictive Transcription in Teleost Green, Red, and Blue Cones. J Neurosci 37:2834–2848.

Fraser B, DuVal MG, Wang H, Allison WT. 2013. Regeneration of Cone Photoreceptors when Cell Ablation Is Primarily Restricted to a Particular Cone Subtype. PLoS One 8:e55410.

Hagerman GF, Noel NCL, Cao SY, DuVal MG, Oel AP, Allison WT. 2016. Rapid recovery of visual function associated with blue cone ablation in Zebrafish. PLoS One 11:1–34.

Hamaoka T, Takechi M, Chinen A, Nishiwaki Y, Kawamura S. 2002. Visualization of rod photoreceptor development using GFP-transgenic zebrafish. Genesis 34:215–220.

Hollingsworth TJ, Gross AK. 2012. Defective Trafficking of Rhodopsin and Its Role in Retinal Degenerations, in: Int Rev Cell Mol Biol. 293:1-44.

Kawamura S, Tachibanaki S. 2008. Rod and cone photoreceptors: Molecular basis of the difference in their physiology. Comp Biochem Physiol A Mol Integr Physiol 150:369–377.

Kennedy BN, Alvarez Y, Brockerhoff SE, Stearns GW, Sapetto-Rebow B, Taylor MR, Hurley JB. 2007. Identification of a Zebrafish Cone Photoreceptor–Specific Promoter and Genetic Rescue of Achromatopsia in the *nof* Mutant. Investig Opthalmology Vis Sci 48:522.

Kennedy BN, Vihtelic TS, Checkley L, Vaughan KT, Hyde DR. 2001. Isolation of a

For Peer Review

zebrafish rod opsin promoter to generate a transgenic zebrafish line expressing enhanced green fluorescent protein in rod photoreceptors.. J Biol Chem 276:14037–43.

Kusser KL, Randall TD. 2003. Simultaneous Detection of EGFP and Cell Surface Markers by Fluorescence Microscopy in Lymphoid Tissues 51:5–14.

Li YN, Tsujimura T, Kawamura S, Dowling JE. 2012. Bipolar cell-photoreceptor connectivity in the zebrafish (Danio rerio) retina. J Comp Neurol 520:3786–3802.

Luo W, Williams J, Smallwood PM, Touchman JW, Roman LM, Nathans J. 2004. Proximal and Distal Sequences Control UV Cone Pigment Gene Expression in Transgenic Zebrafish. J Biol Chem 279:19286–19293.

Makino CL, Wen XH, Michaud NA, Covington HI, DiBenedetto E, Hamm HE, Lem J, Caruso G. 2012. Rhodopsin expression level affects rod outer segment morphology and photoresponse kinetics. PLoS One 7:1–7.

May-Simera H, Nagel-Wolfrum K, Wolfrum U. 2017. Cilia - The sensory antennae in the eye. Prog Retin Eye Res 1–37.

Minamoto T, Shimizu I. 2005. Molecular cloning of cone opsin genes and their expression in the retina of a smelt, Ayu (Plecoglossus altivelis, Teleostei). Comp Biochem Physiol - B Biochem Mol Biol 140:197–205.

Mitchell DM, Stevens CB, Frey RA, Hunter SS, Ashino R, Kawamura S, Stenkamp DL. 2015. Retinoic Acid Signaling Regulates Differential Expression of the Tandemly-Duplicated Long Wavelength-Sensitive Cone Opsin Genes in Zebrafish. PLoS Genet 11:1–33.

Morrow JM, Lazic S, Dixon Fox M, Kuo C, Schott RK, de A Gutierrez E, Santini F, Tropepe V, Chang BSW. 2017. A second visual rhodopsin gene, *rh1-2* , is expressed in zebrafish photoreceptors and found in other ray-finned fishes. J Exp Biol 220:294–303.

Mustafi D, Engel AH, Palczewski K. 2009. Structure of cone photoreceptors. Prog Retin Eye Res 28:289–302.

Ogawa Y, Shiraki T, Kojima D, Fukada Y, Fukada T. 2015. Homeobox transcription factor Six7 governs expression of green opsin genes in zebrafish. Proc R Soc B Biol Sci 282:20150659.

Parichy DM, Elizondo MR, Mills MG, Gordon TN, Engeszer RE. 2009. Normal table of postembryonic zebrafish development: Staging by externally visible anatomy of the living fish. Dev Dyn 238:2975–3015.

Perkins BD, Kainz PM, O'Malley DM, Dowling JE. 2002. Transgenic expression of a GFP-rhodopsin COOH-terminal fusion protein in zebrafish rod photoreceptors. Vis Neurosci

For Peer Review

19:257–264.

Raymond PA, Barthel LK, Curran GA. 1995. Developmental patterning of rod and cone photoreceptors in embryonic zebrafish. J Comp Neurol 359:537–550.

Raymond PA, Colvin SM, Jabeen Z, Nagashima M, Barthel LK, Hadidjojo J, Popova L, Pejaver VR, Lubensky DK. 2014. Patterning the cone mosaic array in zebrafish retina requires specification of ultraviolet-sensitive cones. PLoS One 9:e85325.

Salbreux G, Barthel LK, Raymond PA, Lubensky DK. 2012. Coupling Mechanical Deformations and Planar Cell Polarity to Create Regular Patterns in the Zebrafish Retina. PLoS Comput Biol 8:e1002618.

Samardzija M, Tanimoto N, Kostic C, Beck S, Oberhauser V, Joly S, Thiersch M, Fahl E, Arsenijevic Y, von Lintig J, Wenzel A, Seeliger MW, Grimm C. 2009. In conditions of limited chromophore supply rods entrap 11-cis-retinal leading to loss of cone function and cell death. Hum Mol Genet 18:1266–1275.

Saszik S, Bilotta J. 1999. The effects of temperature on the dark-adapted spectral sensitivity function of the adult zebrafish.. Vision Res 39:1051–8.

Schindelin J, Arganda-Carreras I, Frise E, Kaynig V, Longair M, Pietzsch T, Preibisch S, Rueden C, Saalfeld S, Schmid B, Tinevez J-Y, White DJ, Hartenstein V, Eliceiri K, Tomancak P, Cardona A. 2012. Fiji: an open-source platform for biological-image analysis. Nat Methods 9:676–682.

Shaner NC, Lambert GG, Chammas A, Ni Y, Cranfill PJ, Baird MA, Sell BR, Allen JR, Day RN, Israelsson M, Davidson MW, Wang J. 2013. A bright monomeric green fluorescent protein derived from Branchiostoma lanceolatum. Nat Methods 10:407–409.

Soroldoni D, Hogan BM, Oates AC. 2009. Simple and Efficient Transgenesis with Meganuclease Constructs in Zebrafish, in: Methods in Molecular Biology (Clifton, NJ). pp. 117–130

Sukumaran S, Perkins BD. 2009. Early defects in photoreceptor outer segment morphogenesis in zebrafish ift57, ift88 and ift172 Intraflagellar Transport mutants. Vision Res 49:479–489.

Suliman T, Novales Flamarique I. 2014. Visual pigments and opsin expression in the juveniles of three species of fish (rainbow trout, zebrafish, and killifish) following prolonged exposure to thyroid hormone or retinoic acid. J Comp Neurol 522:98–117.

Suzuki SC, Bleckert A, Williams PR, Takechi M, Kawamura S, Wong ROL. 2013. Cone photoreceptor types in zebrafish are generated by symmetric terminal divisions of dedicated precursors.. Proc Natl Acad Sci U S A 110:15109–14.

For Peer Review

Takechi M, Hamaoka T, Kawamura S. 2003. Fluorescence visualization of ultraviolet-sensitive cone photoreceptor development in living zebrafish. FEBS Lett 553:90–94.

Takechi M, Kawamura S. 2005. Temporal and spatial changes in the expression pattern of multiple red and green subtype opsin genes during zebrafish development.. J Exp Biol 208:1337–1345.

Takechi M, Seno S, Kawamura S. 2008. Identification of cis-acting elements repressing blue opsin expression in zebrafish UV cones and pineal cells. J Biol Chem 283:31625–31632.

Takeuchi Y, Bapary MAJ, Igarashi S, Imamura S, Sawada Y, Matsumoto M, Hur SP, Takemura A. 2011. Molecular cloning and expression of long-wavelength-sensitive cone opsin in the brain of a tropical damselfish. Comp Biochem Physiol - A Mol Integr Physiol 160:486–492.

Tam BM, Moritz OL, Hurd LB, Papermaster DS. 2000. Identification of an Outer Segment Targeting Signal in the COOH Terminus of Rhodopsin Using Transgenic Xenopus laevis 151:1369–1380.

Thummel R, Kassen SC, Montgomery JE, Enright JM, Hyde DR. 2008. Inhibition of Müller glial cell division blocks regeneration of the light-damaged zebrafish retina. Dev Neurobiol 68:392–408.

Tsujimura T, Chinen A, Kawamura S. 2007. Identification of a locus control region for quadruplicated green-sensitive opsin genes in zebrafish. Proc Natl Acad Sci 104:12813–12818.

Tsujimura T, Hosoya T, Kawamura S. 2010. A single enhancer regulating the differential expression of duplicated red-sensitive opsin genes in Zebrafish. PLoS Genet 6:1–10.

Vihtelic TS, Doro CJ, Hyde DR. 1999. Cloning and characterization of six zebrafish photoreceptor opsin cDNAs and immunolocalization of their corresponding proteins.. Vis Neurosci 16:571–585.

Wasfy MM, Matsui JI, Miller J, Dowling JE, Perkins BD. 2014. Myosin 7aa$^{-/-}$ mutant zebrafish show mild photoreceptor degeneration and reduced electroretinographic responses. Exp Eye Res 122:65–76.

Wen XH, Shen L, Brush RS, Michaud N, Al-Ubaidi MR, Gurevich V V, Hamm HE, Lem J, Dibenedetto E, Anderson RE, Makino CL. 2009. Overexpression of rhodopsin alters the structure and photoresponse of rod photoreceptors. Biophys J 96:939–950.

Yates A, Akanni W, Amode MR, Barrell D, Billis K, Carvalho-Silva D, Cummins C, Clapham P, Fitzgerald S, Gil L, Girón CG, Gordon L, Hourlier T, Hunt SE, Janacek SH,

For Peer Review

Johnson N, Juettemann T, Keenan S, Lavidas I, Martin FJ, Maurel T, McLaren W, Murphy DN, Nag R, Nuhn M, Parker A, Patricio M, Pignatelli M, Rahtz M, Riat HS, Sheppard D, Taylor K, Thormann A, Vullo A, Wilder SP, Zadissa A, Birney E, Harrow J, Muffato M, Perry E, Ruffier M, Spudich G, Trevanion SJ, Cunningham F, Aken BL, Zerbino DR, Flicek P. 2016. Ensembl 2016. Nucleic Acids Res 44:D710–D716.

Yin J, Brocher J, Fischer U, Winkler C. 2011. Mutant Prpf31 causes pre-mRNA splicing defects and rod photoreceptor cell degeneration in a zebrafish model for Retinitis pigmentosa. Mol Neurodegener 6:56.

Yokoyama S. 2000. Molecular evolution of vertebrate visual pigments. Prog Retin Eye Res 19:385–419.

Yu C-J, Gao Y, Li P, Li L. 2007. Synchronizing multiphasic circadian rhythms of rhodopsin promoter expression in rod photoreceptor cells. J Exp Biol 210:676–684.

Zou J, Yang X, Wei X. 2010. Restricted Localization of Ponli, a novel zebrafish MAGUK-family protein, to the inner segment interface areas between green, red, and blue cones. Investig Ophthalmol Vis Sci 51:1738–1746.

For Peer Review

Figure 1 – LWS1-G and LWS2-K are detected in OS of PRCs.

A: Schematic illustration of Tg(LWS) (~12 Kb, not to scale). Opn1lw1 and Opn1lw2 are C-terminally fused to mNeonGreen and mKate2, respectively. The regulatory region (orange line) as defined by Tsujimura et al. 2010. The arrow depicts the orientation of the ORF. **B-B'':** Flat-mounted retina of Tg(LWS) with LWS2-K in magenta, LWS1-G in green and brightfield in grey. Asterisks outline the optic nerve of the retina. **C-C'':** Retinal section of an adult Tg(LWS) transgenic fish, ventral to the optic nerve (asterisk in C''). C'' shows the merge of C, C' and DAPI staining (blue). Fluorescence of LWS1-G is marked in green and LWS2-K in magenta. Arrowhead in C'' highlights the outer nuclear layer. **D-D'':** Section of a Tg(LWS) adult retina, showing staining with a red opsin antibody (cyan, D, D'') and fluorescence of LWS2-K (magenta, D', D''). D'' shows the merge of red Opsin antibody staining (cyan), LWS2-K fluorescence (magenta), DAPI staining (blue) and brightfield images. Scale bars: B-C'' 100 μm; D-D'': 10 μm.

Figure 2 – LWS1-G and LWS2-K protein expression during zebrafish retinal development.

A: LWS2-K expression in the pineal gland (arrowhead) of a live zebrafish embryo at 2 dpf. **B-C:** Retinal section of Tg(LWS) at 3 dpf with LWS2-K in magenta and DAPI in blue. White arrowhead points to LWS2-K in OS. **D-D':** Retina of a Tg(LWS) animal of 8.6 mm standard length, showing LWS1-G in green and LWS2-K in magenta. **D'':** Merge of D and D' with DAPI in blue. **D''':** Zoom in on boxed area in D''. **E:** Overview of a pineal gland of a Tg(LWS) larva at 8.6mm SL with LWS2-K in magenta, LWS1-G in green and DAPI in blue. **E'-E'':** Blow-up of boxed area in E. **F:** RT-PCR results for WT (lane 2 to 5) and TG(LWS) (lane 6 to 9) littermates. cDNA was prepared from 3 dpf, 5 dpf, 8.6 mm SL larvae and adult retinas (ad). WT amplicons were obtained using *opn1lw1* specific primers. TG(LWS) PCR products were amplified using mNeonGreen specific primers. Lane 1: marker. Scale bars: A – 1mm; B, D-F and G -100 μm; B', F', G'-G'': 10 μm; C: 5 μm.

Figure 3 – No obvious changes were detected in OS size of Tg(LWS) PRCs.

A: PRC of Tg(LWS)/Tg(Ola.Actb:Hsa.HRAS-EGFP)^{vu119} at 3 dpf showing mKate2 in magenta and GFP at the plasma membrane in green. OS height (h, dotted line) was calculated by measuring the distance from the mid- point at the base of the OS until the tip of the OS. OS width (w, full line) was calculated by measuring the length of the OS base. **B-C:** OS

For Peer Review

height (B) and width (C) were measured in both Tg(LWS)/ Tg(Ola.Actb:Hsa.HRAS-EGFP)^{vu119} (white box) and Tg(Ola.Actb:Hsa.HRAS-EGFP)^{vu119} stained with red opsin antibody (grey box with dots) at 3 dpf, 4 dpf and 5 dpf. Measurements are represented in box plots with calculated minimum, 25th percentile, mean, 75th percentile and maximum. The x-axis shows the age of the embryos in days post fertilization (dpf) and the y-axis shows the length measured in μm. For all time-points and different transgenic lines at least 3 independent samples were used and 20 measurements were carried out. Statistical significance was calculated by a one-way ANOVA followed by Tukey's multiple comparison test. No statistically significant differences were observed.

Figure 4 –LWS2-K signals in embryonic and adult retinas.

Retinal sections of Tg(LWS) with LWS2-K in magenta and DAPI in blue. **A-B'':** Vesicle-like structures are present in the IS of LWS2-K positive cells. **A-A'':** Retinal sections of adult Tg(LWS). **B-B'':** Retinal sections of Tg(LWS) at 3 dpf. **C-C'':** Retinal sections of Tg(LWS) with bright-field image in grey scale. Asterisks indicate the LWS2-K positive signal in the retinal pigment epithelium, which is likely due to the pigment present in these cells. Scale bars: A-C'' 10 μm.

For Peer Review

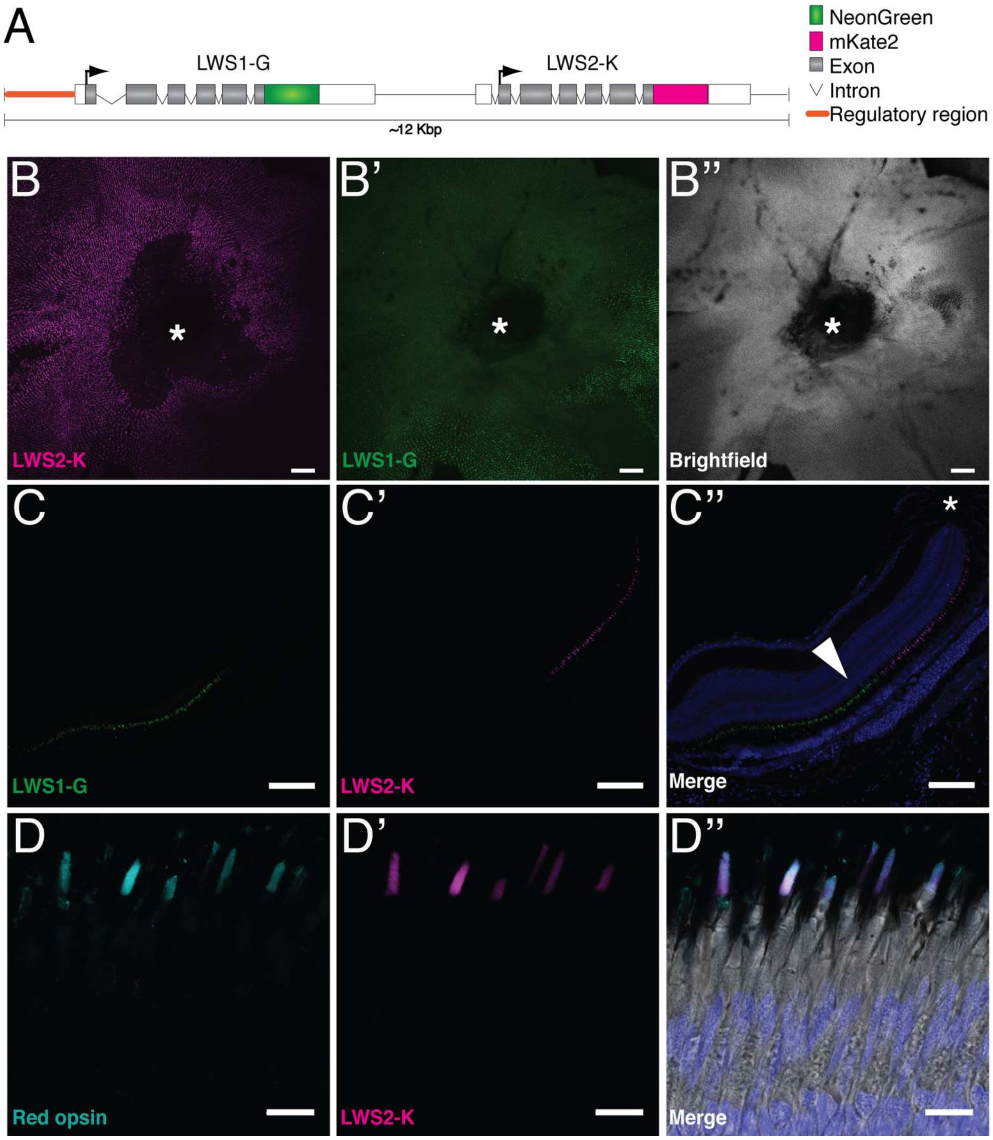

Fig. 1

198x224mm (300 x 300 DPI)

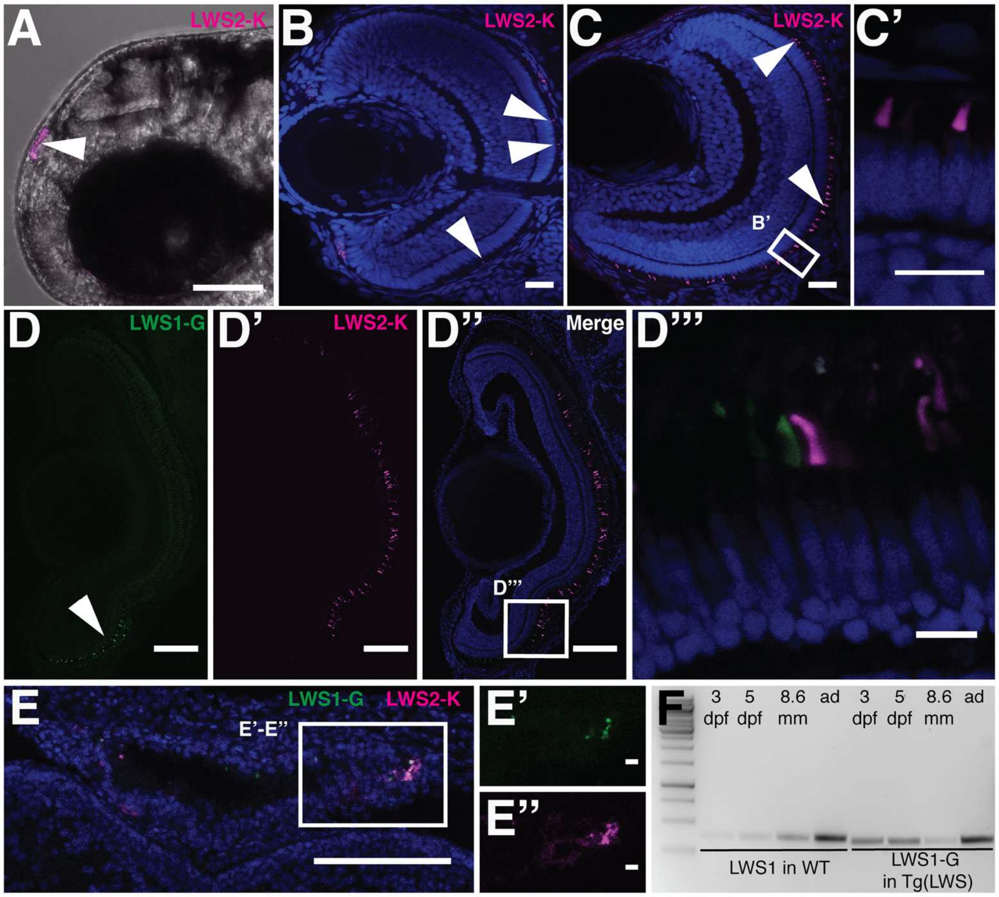

Fig. 2

155x139mm (300 x 300 DPI)

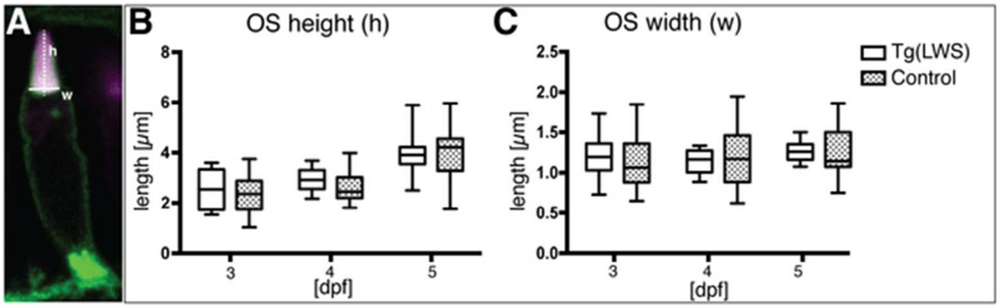

Fig. 3

53x16mm (300 x 300 DPI)

For Peer Review

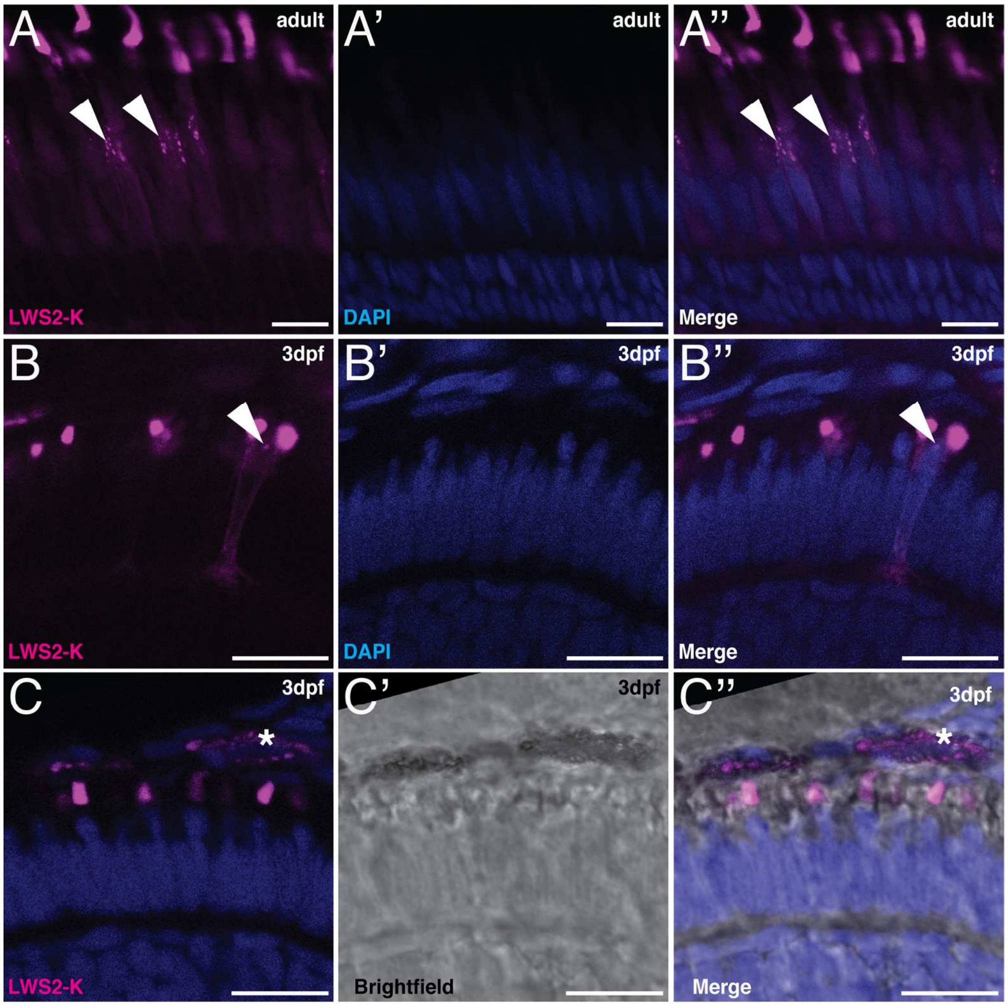

Fig. 4

172x172mm (300 x 300 DPI)
